# Sheep erythrocyte potassium locus highlights MYADM family regulation of electroneutral potassium chloride cotransporters (KCC)

**DOI:** 10.1186/s12864-026-13029-7

**Published:** 2026-06-16

**Authors:** Michael V. Gonzalez, Michelle R. Mousel, David R. Herndon, Alisha T. Massa, Codie J. Durfee, Donald P. Knowles, J. Bret Taylor, Holly L. Neibergs, Christopher M Seabury, Stephen N. White

**Affiliations:** 1 Animal Disease Research Unit, Agricultural Research Service, Department of Agriculture, Pullman, WA 99164 USA; 2 Department of Veterinary Microbiology and Pathology, Washington State University, Pullman, WA 99164 USA; 3 School for Global Health, Washington State University, Pullman, WA 99164 USA; 4 Current address: Genus Research and Development, DeForest, WI 53532 USA; 5 Veterinary Medical Research & Development, Pullman, WA 99163 USA; 6 U.S. Sheep Experiment Station, Agricultural Research Service, Department of Agriculture, Dubois, ID 83423 USA; 7 Department of Animal Sciences, Washington State University, Pullman, WA 99164 USA; 8 Veterinary Medicine and Biomedical Sciences, Texas A&M University, College Station, TX 77843 USA; 9 Current address: Poultry Microbiological Safety and Processing Research, U.S. National Poultry Research Center, U.S. Department of Agriculture, Athens, GA 30605 USA

**Keywords:** MYADM, MYADMF, Livestock, Erythrocyte, Hypertension, Sheep, Goat, Cattle, Pig, KCC, KCC3A, N-terminal peptide, AlphaFold3, Potassium chloride co-transport, GWAS

## Abstract

Some domestic sheep have distinct low (LK) or high intracellular potassium (HK) erythrocytes with demonstrated differences in potassium chloride co-transport. Erythrocyte potassium concentration is inherited as a single locus trait, although responsible genetic variant(s) have not been identified. Herein, genome-wide association identified a large, artiodactyl-specific tandem duplication of the *MYADM* gene family locus as highly associated (*P* = 1.1 × 10^− 63^) with erythrocyte intracellular potassium concentration. The full tandemly expanded *MYADMF* locus included 37 genes in sheep, 33 genes in goats, 52 genes in cattle, and 15 genes in domestic swine, but no comparable tandemly expanded locus in horses. Fine mapping in an expanded sheep population showed maximal association with erythrocyte potassium (*P* < 10^− 99^) for *MYADMF15A1*,* MYADMF1G1*,* MYADMF1H1*, *MYADMF3A1*, *MYADMF1B3*, and *MYADMF13C1*. Prior data showed higher potassium chloride co-transport in sheep LK than HK erythrocytes, and the present data, for the first time, implicate one or more *MYADMF* genes in the regulation of potassium chloride co-transport. Since there are many fewer *MYADM* genes in humans, we hypothesized that a simpler interaction might exist with the gene family namesake *MYADM*, which has been linked to human hypertension in multiple studies. Alphafold3 protein models suggest *MYADM* interacts directly with *KCC3* to stabilize its N-terminal regulatory peptide in the inhibitory position. While the complexity of the *MYADMF* expanded repeat locus makes it more difficult to draw conclusions about causation, it is possible that divergent alleles such as *MYADMF1G1 and MYADMF1B3*, or possibly *MYADMF15A1*, cause the low potassium phenotype through interaction with *KCC3* and effects on the N-terminal regulatory peptide. Cumulatively, these data identify an expanded *MYADMF* locus as the major controller of sheep erythrocyte potassium concentration and suggest direct interaction of *MYADM* proteins with *KCC* transporters may regulate their function in diverse contexts. Additional work is needed to confirm structural predictions and explore functional implications.

**Supplementary Information:**

The online version contains supplementary material available at 10.1186/s12864-026-13029-7.

## Introduction

Sheep red blood cells (RBCs) fall into two distinct classes: high (HK) or low (LK) intracellular potassium [1–3]. Cation transport studies using HK and LK cells described the first reports of chloride-dependent K+ efflux [4, 5], which was later recognized as the function of electroneutral K-Cl cotransporters that are important in cell volume homeostasis and hypertension [6–10]. In some studies, the intracellular potassium dimorphism has been shown to be associated with wool quality and growth [11]. The inheritance of high and low intracellular potassium levels in RBC has been reported to be a simple single-locus trait [12], with the assumption that differences in potassium levels are due to a mutation in one of the K-Cl cotransporters [13]. However, the underlying basis of the physiologically distinct LK and HK cell types has not been elucidated [13].

High and low potassium erythrocytes have specific serologically determined antigens on their cell surface. The HK cells are homozygous for the M blood-group antigen [14, 15], whereas the LK cells were homozygous or heterozygous for the L antigen [15–17]. Additionally, the L antigen was shown to act as an inhibitor of active potassium transport in red blood cells, as treatment with anti-L antibody caused a 4-to 5-fold increase in potassium transport [18]. Conversely, treatment of red cells with anti-M had no effect [16, 19]. Together, this argued that the inhibition of potassium transport in LK red blood cells occurs, at least in part, due to antibody-exposed potassium transporter site(s) on the cell surface.

The K-Cl co-transporters (KCC proteins) are recognized as accounting for the observed features of LK sheep erythrocytes [8, 13]. This protein subfamily is encoded by *SLC12A3-SLC12A6* genes that produce KCC1-KCC4 proteins, respectively [6, 20–22]. The chloride-dependent potassium efflux enabled by KCC proteins has long been a hallmark of LK sheep erythrocytes [5]. An additional feature of LK and HK erythrocytes is the strikingly different intracellular sodium concentrations, with LK cells having high sodium levels and HK cells having low sodium levels [23, 24]. This may be due to the functional association of KCC3 with the Na+, K+-ATPase, which KCC3 up-regulates [25, 26] and the expression of KCC1 and KCC3 in sheep erythrocytes [27]. Sheep LK and HK erythrocytes were fundamental to the discovery of KCC proteins. Indeed, following the KCC discovery, many in the field believed that the sheep genetic variant responsible for the LK/HK phenotype would be found in either *SLC12A4* or *SLC12A6*, the genes that produce the primary cotransporter proteins KCC1 and KCC3 [13]. However, a specific search for genetic variants in these genes did not yield any that could account for the large LK/HK phenotype [28]. The specific characteristics of the mutation responsible for the LK/HK difference in erythrocytes are: (1) it is composed of a single locus, (2) it has alleles at high frequency in multiple sheep breeds around the world [23, 24, 29], (3) the alleles control potassium concentrations for LK and HK cells [23], (4) it possesses a dominant or incomplete dominant mode of inheritance of LK and HK alleles [12, 30, 31], (5) similar segregating alleles exist among for LK and HK cells in diverse ruminants (including goat, cow, yak, water buffalo, wildebeest, Speke’s gazelle, etc.) [32–37], and (6) there is antibody recognition [16, 18, 38] of LK/HK expressed as cell surface differences. These characteristics define an important genetic variant that has thus far remained elusive [39].

Transmembrane cation transport is a critical mechanism for cellular volume maintenance in mammalian cells, including RBCs [1, 40]. We hypothesized that cation transport in LK and HK sheep RBC are related to red cell volume differences observed with a previously identified divergent artiodactyl *MYADM-like* repeat (*MYADMF1F1*) [41]. To test this hypothesis and identify other genes that might be associated with RBC intracellular potassium and sodium concentration in sheep, we performed a GWAS utilizing a high-density SNP chip with over 500,000 single-nucleotide polymorphisms (SNPs) [42] in four breeds of domestic sheep. The GWAS was followed by fine mapping to identify putative causal mutations within the associated loci. To our knowledge, this is the first report of specific loci associated with erythrocyte intracellular cation levels in an artiodactyl species.

The myeloid-associated differentiation marker (*MYADM*) [43] gene family is small, with only two members in humans and most mammals (*MYADM* and *MYADM-like 2*). MYADM possesses two MARVEL domains [44], and *MYADM* transcription has been shown to correlate with human blood pressure and hypertension [45–47]. *MYADM* is the target of miR-182-3p, and both have been implicated in regulating hypertension [48, 49]. However, important questions remain about how *MYADM* influences blood pressure and hypertension.

AlphaFold and its successor algorithms have revolutionized protein structure prediction by achieving high accuracy that often rivals benchwork experiments [50–52]. Such tools have been used to gain notable insights into numerous processes [53–56]. AlphaFold-Multimer was an earlier version that has been used to achieve insights, including protein interactions [57, 58]. Alphafold3 has recently substantially improved and expanded the range of applications for protein structure prediction, particularly for protein-protein interactions [52]. We applied Alphafold3 to test whether there might be direct interaction between MYADM and KCC proteins, used structural insights to propose a key mechanism, and then broadened mechanistic insights to MYADMF proteins more generally.

## Materials and methods

### Ethics statement

All animal care and handling procedures were reviewed and approved by the Washington State University Institutional Animal Care and Use Committee (Permit Numbers: 04421 and 04594) and/or by the U.S. Sheep Experiment Station Animal Care and Use Committee (Protocol Numbers: 04–14, 10 − 07). All efforts were made to minimize any discomfort during the blood collection process. No animals were euthanized for this study.

### Cation quantitation

Whole blood was collected via jugular venipuncture from 307 ewes of Columbia (*n* = 30), Polypay (*n* = 91), Rambouillet (*n* = 106), and Suffolk (*n* = 80) breeds, aged 1–10 years, from the USDA, Agricultural Research Service, US Sheep Experiment Station near Dubois, Idaho, into lithium heparin-coated vacutainer tubes as previously described [28, 59]. These animals were managed similarly but bred separately in purebred groups.

Blood tubes were inverted to mix thoroughly immediately after collection. Two milliliters of whole blood was centrifuged for one minute at 10,000xg to achieve red blood cell pack. Plasma and buffy coat were discarded. The RBC pack was stored at 6 °C overnight. The RBC pack was vortexed thoroughly and allowed to return to room temperature. One hundred µL of RBCs was added to 9.9 mL (1/100 dilution) of 1000 µg /mL cesium chloride using a wide-bore tip. Dilutions of RBCs were made in triplicate.

Cation content of RBCs was measured using a Varian Spectra 220 Atomic Absorbance Spectrometer. Wavelength and slit were adjusted according to the manufacturer’s instructions (Varian, Palo Alto, CA). Each cation recording was an average of three replicates. A 1,000 µg/mL cesium chloride solution was used as a rinse between animal samples. Samples that tested outside the equipment range were diluted a second time to achieve cation readings within range. Potassium standards were prepared at concentrations of 0.25, 0.5, 1, 3, and 4 µg/mL from a 1000 µg/mL stock solution diluted in 1000 µg/mL cesium chloride. Sodium standards were prepared at concentrations of 5, 10, 20, 30, and 50 µg/mL from a 1000 µg/mL stock solution diluted in 1000 µg/mL cesium chloride. Units of cation phenotypes are µg /mL.

### Genotyping with high density illumina ovine SNP chip

A subset of 104 sheep were genotyped at high density, including Columbia (*n* = 10), Polypay (*n* = 22), Rambouillet (*n* = 13), and Suffolk (*n* = 59) breeds, aged 1–7 years. DNA was isolated using the Invitrogen GeneCatcher™ gDNA Blood Kit (3–10 ml) according to the manufacturer’s instructions (Life Technologies, Carlsbad, CA). Genotyping services were provided by Geneseek Inc. (Lincoln, NE) using the Ovine Infinium HD SNP Beadchip (Illumina Inc., San Diego, CA) with a set of 603,350 SNPs designed by the International Sheep Genome Consortium [42].

### BAC assembly/sequencing of *myadmf* alternate allele

Target PCR primers (Table S1) designed to amplify the *MYADMF* region were used for screening a sheep genomic library by superpool and pool as previously described [60]. BAC DNA from the target clone was extracted using standard alkaline lysis methods and submitted to the Genomics Core lab at Washington State University (Pullman, WA). A 10 kb target size library was constructed using the SMRTbell Template Prep Kit (Pacific Biosciences) and sequenced with a single SMRT cell, employing the P4 binding kit and C2 reagent chemistry. De novo assembly was performed using the Hierarchical Genome Assembly Process (HGAP) version 3 workflow, as integrated into the SMRT analysis package, with a minimum seed read length of 3000 bp.

### *Ovis aries MYADMF* alternate and reference (ARS-UI Ramb v2.0) allele comparison

*MYADMF* open reading frames (ORFs) were identified in the BAC alternate allele sequence using sheep *MYADM* mRNA (XM_004023524.2) for BLAST analysis at the nucleotide level. Initial relationships among the alternate and reference allele *MYADMF* gene repeats were ascertained by amino acid sequence identity and aligned using the MUSCLE algorithm within the MEGA software package [61]. From this alignment, a maximum likelihood phylogenetic tree was constructed using 500 bootstraps in MEGA. This phylogenetic tree was used to determine the closest common ancestor(s) of *MYADMF* genes between the sheep reference and alternate assemblies, using a threshold of greater than 60% from the bootstrap analysis.

### Re-sequencing of alternate and reference allele homozygotes

Seven total *MYADMF* homozygous sheep were sequenced, including three Columbia, two Polypay, and two Rambouillet. These animals were selected using their genotype and RBC phenotype as selection criteria (i.e. two reference allele homozygotes showed increased mean corpuscular hemoglobin concentration (MCHC), decreased mean corpuscular volume (MCV), and five alternate allele homozygotes showed phenotypes within normal reference ranges. Genomic DNA was extracted (Invitrogen GeneCatcher gDNA Blood Kit, Waltham, MA) and quantified (Nanodrop, Waltham, MA). Sequencing libraries were constructed at the University of Idaho Genomics Resource Core (IBEST; Moscow, Idaho). Briefly, genomic DNA was sheared using a Covaris M220 (Woburn, MA), and automated libraries were prepared with the Apollo 324 (Wafergen Biosystems, Fremont, CA) system, with a target insert size of 520 bp. Sequencing was performed at the University of California, Irvine Genomics and High-Throughput Facility using an Illumina HiSeq instrument (San Diego, CA), set to generate paired sequence reads with one full lane per animal. Sequences were mapped to the *Ovis aries* genome sequence (OAR v.2.0) using bowtie2 [62] with the following parameters: insert size set to 1000 bp, paired-end alignment requirement, and default ambiguous match requirement. Alignment files were converted to sorted BAM files using SAMtools [63] and viewed with the Integrative Genomics Viewer (IGV, version 2.3, San Diego, CA) [64] or imported into CLC Genomics Workbench (version 8.0, Qiagen, Redwood City, CA) for variant calling.

### Identification of putative mutations and additional polymorphisms

Two methods were used for variant calling between the reference (ARS-UI_Ramb v.2.0) and alternate alleles (BAC Sequence) at the *MYADMF* expanded loci in domestic sheep. The first method utilized the CLC Genomics Workbench Suite (Qiagen Aarhus A/S, Germantown, MD), where variants were identified using the Basic Variant Detection tool with a ploidy setting of two. Additional settings included: ignoring positions with coverage greater than 100,000x, not ignoring broken pairs, a minimum of 5x coverage, and a read count of one with an 80% frequency.

The second method involved duplicate, blind manual variant calling within *MYADMF* ORFs using the IGV and sheep re-sequencing data. Manual variant calls were performed by two separate technicians, and the two lists were reconciled after individual analysis. Open reading frames within the *MYADMF* alternate region were defined within the hybrid reference using BLAST. Single-nucleotide variants and short indels were called when the variant was present on 100% of reads in two reference allele homozygotes and > 50% in five alternate allele homozygotes using the BAC for additional comparison.

### Comparative analysis of *MYADMF* loci in other animal species

Preliminary analysis determined that *MYADMF* gene sequences in the expanded repeat locus were more similar within species than between species. *MYADM* gene mRNA sequences from cow (*NM_001075252.2*), sheep (*XM_004023524.2*), goat (*XM_005692928.1*) pig (*XM_005664815.1*), horse (*XM_005596496.1*), human [*NM_001020818.2*], dog [*XM_005616122.1*], southern white rhinoceros [*XM_004439043.1*], Przewalski’s horse [*XM_008528176.1*] and mouse [*NM_001093764.1*] were used to perform nucleotide and translated BLAST searches of the respective genomes (cow: ARS-UCD1.2; sheep ARS-UI Ramb v2.0; goat CHIR_1.2; pig Sscrofa 11.1; horse EquCab 2.0; human GRCh38.p2; dog CanFam3.1; rhino CerSimSim1.0; mouse GRCm38.p3; Przewalski’s horse Burgud [65–79]. BLAST hits were compiled, noting the genomic position, including the open reading frame, start, stop, and orientation. For the *MYADMF* nomenclature, a 65% identity threshold was used for at least one member of a family (i.e., not all members had to exceed this threshold with respect to all other individual members). In cases where multiple subfamilies exceeded this threshold, the best identities determined the nomenclature choice. Orthologs between *MYADMF* repeat loci in different species were defined by > 85% amino acid identity over 90% of ORF length for the shorter member of any pairwise comparison.

### Genotyping of expanded cation phenotype sheep flock

The complete group of 307 animals, comprising Columbia (*N* = 30), Polypay (*N* = 91), Rambouillet (*N* = 106), and Suffolk (*N* = 80) breeds, aged 1–10 years, had intraerythrocytic cation phenotypes collected. This expanded population was used in further genotype testing for fine mapping purposes.

Multiple methods were used to genotype genetic variants in *MYADMF* ORFs. These included TaqMan^®^ Genotyping Assays (Table S2) and allele-specific PCR (Table S1). TaqMan assays were performed according to the manufacturer’s instructions (Applied Biosystems, Foster City, CA). All PCR was performed using Accuprime™ Supermix II master mix per manufacturer’s instructions (Invitrogen, Carlsbad, CA, USA). PCR was temperature-optimized for the annealing and extension phases as described previously [41]. Allele-specific PCR targeting the divergent artiodactyl *MYADM-like* repeat (*MYADMF1F1)* was performed as described previously [41].

Because of the high number of genetic variants identified in *MYADMF* genes, these regions were sequenced in a population of 307 domestic sheep of Columbia (*N* = 30), Polypay (*N* = 91), Rambouillet (*N* = 106), and Suffolk (*N* = 80) breeds. PCR began with an initial denaturation step at 95 °C for 10 min, followed by 40 cycles of 95 °C for 30 s, 63 °C for 30 s, and 68 °C for 90 s. All reactions were performed using Accuprime™ Supermix II master mix according to the manufacturer’s instructions (Invitrogen, Carlsbad, CA). Samples were then submitted to the Genomics Core Lab at Washington State University (Pullman, WA), where amplified products were sequenced by standard dideoxy nucleotide analysis using multiple primers (Table S1). Since pilot data indicated that causal variants would be common in this population, only variants identified with a minor allele frequency greater than 5% were included for further analysis.

### Statistical Analysis

#### Genome-wide association analysis

Red blood cell cation phenotypes were analyzed using analytic models similar to those previously described [41, 59]. Specifically, linear models were used to account for breed (as binary-coded categorical variables), animal age in years (as a covariate), and the SNP minor allele. For all analyses, PLINK screening criteria for continued analysis included missingness by individual (individuals missing more than 10% of genotyping calls), missingness by marker (SNP markers missing more than 3% of genotyping calls), minor allele frequency (MAF > 2%), (*P* > 0.000001). The PLINK sheep chromosome setting focused analyses on autosome data, and unfortunately precluded use of X chromosome data. In accordance Wellcome Trust work, genome-wide significance was defined by nominal *P*≤5 × 10^− 8^ [80–82], and genome-wide suggestive was defined as *P*≤1 × 10^− 5^ [52, 68]. Recent work has suggested a relaxed threshold of 5 × 10^− 7^ may improve discovery [83], but we preferred a more conservative genome-wide significance level, noting that many important results have been identified at *P*<5 × 10^− 8^ [84–86].

Association models used included additive, genotypic two-degree-of-freedom, dominant, or recessive modes of inheritance. Manhattan and quantile-quantile plots were generated using R [87]. SAS 9.2 (SAS Institute, Cary, NC) was used to run similar statistical models to those used with PLINK software to obtain the largest adjusted genotypic mean differences for each RBC cation phenotype as a measure of allele effect size.

#### Fine mapping association analysis of putative mutations and genetic variants

Association testing between genetic variants and intraerythrocytic cation concentration phenotypes were performed using a mixed model with SAS 9.2. The model included cation phenotype as the dependent variable in each analysis. Fixed effect independent variables included breed as a categorical predictor, age in years as a covariate, and genotype. Sire was included as a random effect nested within breed. These models provided F-statistics for fine mapping.

### Alphafold3 Structural Models

Alphafold3 models were constructed using AlphaFold Server [52]. In each case, KCC proteins were modeled as dimers based on known function in that configuration [88, 89]. For the broad look at MYADM interactions, human *MYADM* primary sequence was taken from NP_001277122.1, *KCC3A* from NP_001352017.1, and *KCC3B* from NP_005126_1. Additional KCC primary sequences included: *KCC1* NP_001139433.1, NP_001139435.1, and NP_001139436.1, *KCC2* NP_065759.1, and *KCC4* NP_006589.2. Ovine *MYADM* primary sequence was from XP_014956187.2, and *KCC3A* was from XP_012036524.1.

For the sheep erythrocyte potassium function, we used ovine *KCC3A* from XP_012036524.1 since *KCC3A* is known to be the predominant isoform in erythrocytes [90, 91]. MYADMF protein primary sequences were taken from the ovine expanded repeat locus, which was from the Texel reference genome OARv4 (GCA_000298735.2) and from our BAC sequence, which had opposite genotypes for SNPs: oar3_OAR18_19267427 = rs410571717, oar3_OAR18_19342055 = rs428719951, oar3_OAR18_19354404 = rs424845901, and oar3_OAR18_19381535 = rs419187285. In each case, the open reading frames for each *MYADMF* gene were identified using ORF-Finder [92].

Models were visualized on the AlphaFold Server, and also using ChimeraX 1.9 [93]. Interface residues were obtained from ChimeraX 1.9 using the interfaces function with a MYADM-KCC interaction edge by selecting all contact residues from both proteins and listing to log with the command “info residues sel” [93].

## Results

### Genome-wide association with intracellular erythrocyte cation phenotypes

**Fig. 1:**
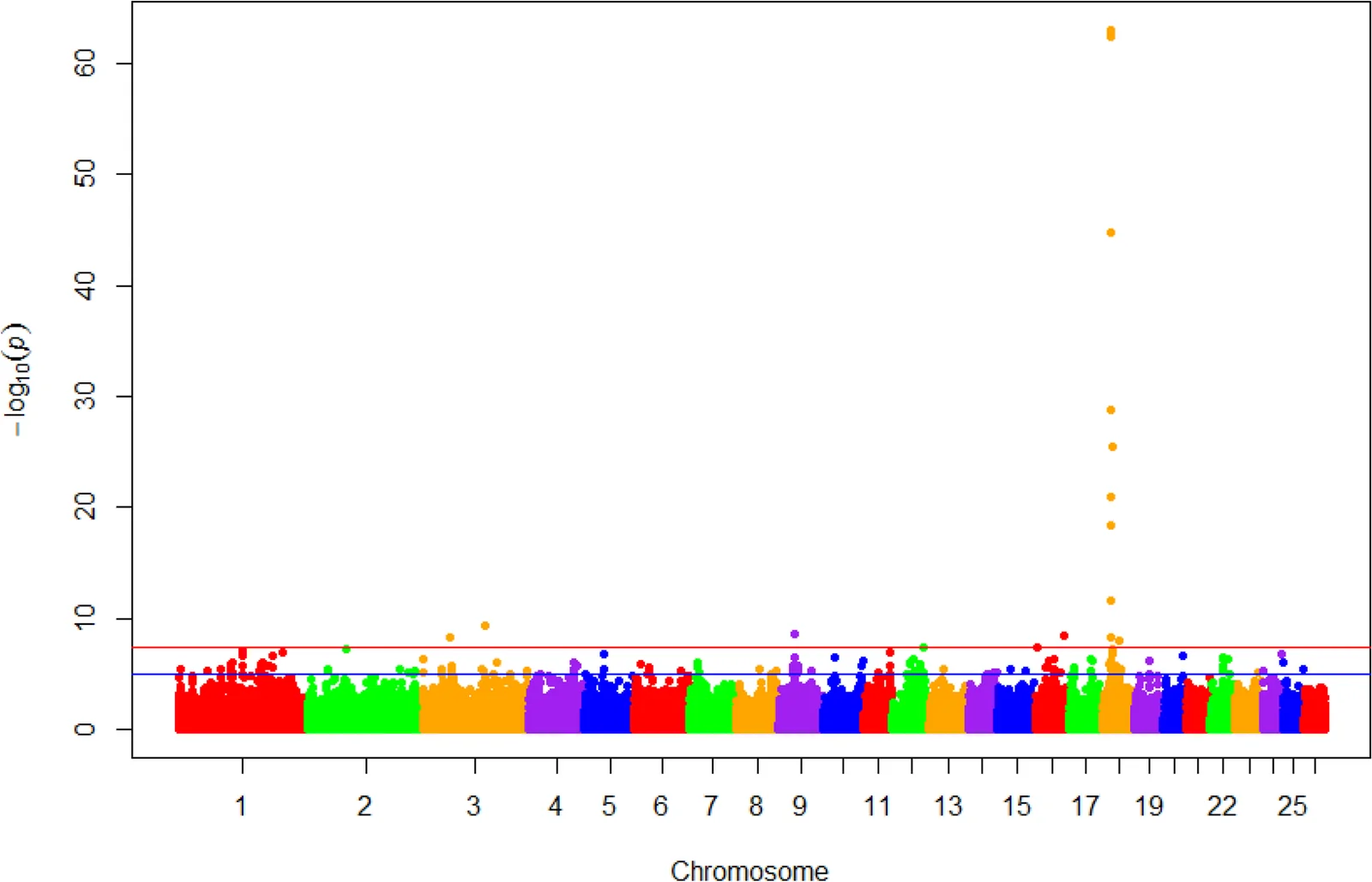
Manhattan plot of erythrocyte potassium concentration. The red line signifies genome-wide significance (*P*=5 × 10^− 8^), and the blue line signifies genome-wide suggestive association (*P*=1 × 10^− 5^)

Three RBC phenotypes were analyzed: potassium concentration, sodium concentration, and sodium-to-potassium ratio. Animal age was not associated with potassium concentration, sodium concentration, or the sodium/potassium ratio (all *P* > 0.05); therefore, age was not retained in the model. After quality screens for genotyping call rate by individual and by SNP, SNP minor allele frequency, and failure of SNPs to meet Hardy-Weinberg equilibrium, the total SNPs analyzed for the cation marker-phenotype GWAS was 524,208. A Manhattan plot of the erythrocyte potassium results is shown in Fig. 1. Additional Manhattan plots for erythrocyte sodium concentration and the sodium-to-potassium ratio are shown in Figures S1 and S2. A total of 25 SNPs showed genome-wide significant associations (*P* < 5 × 10^- 8) with at least one of the investigated phenotypes (Tables S3-S5); 14 SNPs achieved genome-wide significance in all three RBC cation phenotypes. A summary of *MYADMF* loci is provided in Table S6-9 for context on these findings. Sixteen of the 25 SNPs reaching genome-wide significance mapped to chromosome 18 of the domestic sheep genome, containing an array of *MYADMF* genes. Of these, four SNPs showed maximal (and exactly equivalent) association with all intraerythrocytic cation phenotypes on chromosome 18 (oar3_OAR18_19267427 = rs410571717, oar3_OAR18_19342055 = rs428719951, oar3_OAR18_19354404 = rs424845901, and oar3_OAR18_19381535 = rs419187285). Upon further investigation, genotypes at these four peak SNPs were found to be in strong linkage disequilibrium with divergent artiodactyl *MYADMF* repeat genotypes (*MYADMF1F1*) previously implicated in affecting RBC and production traits in domestic sheep (D’=0.97, r^2^ = 0.87).

Animals inheriting two copies of the reference allele showed markedly increased RBC intracellular potassium (HK) and decreased intracellular sodium as well as Na/K ratio compared to heterozygotes and alternate allele homozygotes (Table 1). This genomic interval includes *MYADMF* members: *MYADMF1I1*,* MYADMF15A1*,* MYADMF1G1*,* MYADMF1H1*,* MYAMDF3A1*,* MYADMF1B3*,* and MYADMF13C1* in ARS-UI_Ramb v.2.0. Other SNPs associated with at least one cation phenotype were found on chromosomes 1, 2, 3, 12, 14, and 16.

**Table 1:** Genotypic means for cation phenotype peak SNPs

Phenotype	Ref/Ref = HK (*µg/ml)	Ref/Alt = LK (*µg/ml)	Alt/Alt = LK (*µg/ml)	Nominal *P*-value
RBC K	2465.16	495.43	326.16	1.11E-63
RBC Na	1059.44	2501.62	2653.65	1.46E-32
RBC NaK	0.43	5.05	8.14	6.39E-21

* µg/ml of RBC lysate

### BAC sequencing of alternate (LK) allele

After BAC sequencing and assembly, the resulting contig was ~ 295 kb in length. This assembly had an average coverage of 623X, with a minimum coverage of> 500X throughout its entire length. The average consensus concordance between seed reads and non-seed sequence reads was 99.96% for all assemblies. Additionally, BAC end sequencing confirmed the position with strong BLAST hits at 19,068,232 and 19,410,352 of the reference domestic sheep genome ARS-UI Ramb v2.0 [77].

The ~ 295 kb BAC contig was found to contain 15 *MYADMF* ORFs relative to the ARS-UI_Ramb v.2.0 reference assembly corresponding to *MYADMF* members: *MYADMF11A1*,* MYADMF12A1*,* MYADMF2C1*,* MYADMF13A1*,* MYADMF1F4*,* MYADMF14A1*,* MYADMF13B1*,* MYADMF1B2*,* MYADMF1I1*,* MYADMF15A1*,* MYADMF1G1*,* MYADMF1H1*,* MYAMDF3A1*,* MYADMF1B3*,* and MYADMF13C1*. Out of this list of genes, the sheep reference sequence contains only one *MYADMF* ORF, an apparent full-length *MYADMF* member (*MYADMF1F1*), which appears deleted in the BAC assembly (alternate allele). Additionally, 6 *MYADMF* ORFs appear larger (> 100 bps) in the alternate allele relative to the reference, specifically, *MYADMF* members *MYADMF13A1*,* MYADMF15A1*, *MYADMF1G1*, *MYADMF1H1*, *MYADMF3A1*,* and MYADMF1B3*. We detected a sequence with shared identity with exon 3 of *ISG20*, an upstream gene on chromosome 18, as being intergenic between *MYADMF* members *MYADMF1H1* and *MYADMF3A1* within the association peak. However, no other sequences sharing identity with exons of *ISG20* were found in the comparative analysis.

### Comparative analysis of *myadmf* region across mammalian radiation

*MYADMF* ORFs were identified in additional species, at the nucleotide level, using the species-specific *MYADM* ORF. A comparative map illustrates the comparative positions of *MYADMF* genes from sheep, goat, and cow (Figure S3). A phylogenetic tree including *MYADMF* genes from sheep, goat, and cow was constructed (Figure S4). Additionally, orthologs for *MYADM* and *MYADML2* were identified in all mammal species analyzed. *MYADMF* genes were identified in human and dog but showed no conserved synteny with the artiodactyl expansion. *MYADMF* repeat loci were identified in artiodactyls, including cow, sheep, goat, and pig, but not in representative perissodactyls (horse, Przewalski’s horse, or rhinoceros) or other mammals. Comparative analysis identified clear orthologues of the sheep *MYADMF* genes in ruminants (cow and goat), but not in non-ruminants (pig), where the sequences were more distantly related.

The goat (*Capra hircus*) reference genome (v.1.2) contains 33 *MYADMF* sequences located on chromosome 21. Additionally, a 628 kb locus containing 52 *MYADMF* sequences was identified on cow (*Bos taurus*) chromosome 21 (ARS-UCD1.2). Pig (*Sus scrofa*) also showed evidence of a *MYADMF* expansion on chromosome 7; however, after further investigation, we noted that these fewer *MYADMF* members (15 in total) appeared to be more distantly related relative to sheep, cattle, and goat, with many fewer simple 1:1 ortholog relationships.

Currently annotated *MYADMF* genes were investigated in each respective artiodactyl genome (ARS-UI_Ramb v.2.0), goat (1.2), cow (ARS-UCD1.2), and pig (Sus scrofa11.1). A number of the *MYADMF* genes have been annotated with a “LOC” or “novel” designation followed by a unique numeric identifier in NCBI and Ensembl, respectively, while others remain unannotated (NCBI RefSeq release 72, Ensembl Release 82), indicating that a published symbol is not currently available for these *MYADMF* sequences. A total of 138 *MYADMF* ORFs associated with homologous tandem repeat regions have been identified in artiodactyls. To better organize the *MYADMF* genes in artiodactyls, amino acid percent identity was used to organize ORFs into a numbered family/subfamily structure similar to the CYP olfactory receptor genes [94]. In this naming scheme, ORFs sharing greater than or equal to 60% pairwise identity at the amino acid level are given a unique numeric identifier. If the pairwise identity is greater than or equal to 70%, the *MYADMF* ORFs are organized into a letter-designated subfamily.

### Variant calling in sheep re-sequence data

A total of 1,287 variants were identified using a combination of automated and manual criteria. This included 41 short (up to 11 bps in length) insertions or deletions (indels), 77 multiple nucleotide variants (MNV), and 1,168 single nucleotide variants (SNV). Within *MYADMF* ORFs, a total of 98 short variants were identified (Table S10). The focus of this analysis is on 69 variants found within defined ORFs identified in the potassium GWAS peak between SNP oar3_OAR18_19,226,336 and oar3_OAR18_19,395,896 that altered amino acid composition encoded by *MYADMF* (Table S10). This included 1 single nucleotide variant (SNV) in *MYADMF1I1*, 24 SNVs and 1 short insertion-deletion (indel) in *MYADMF15A1*, 20 SNVs in *MYADMF1G1*, 5 SNVs in *MYADMF1H1*, 7 SNVs and 1 multiple nucleotide variant (MNV) in *MYADMF3A1*, and 8 SNVs in *MYADMF13C1*. Additionally, 60 synonymous variants were identified in the genotyping by sequencing data, which were also included in the subsequent analysis of the expanded population.

### Genotyping in expanded animal cohort

A total of 129 genetic variants were genotyped in 307 domestic sheep from 4 breeds using a combination of Taqman genotyping assays, allele-specific PCR assays, and genotyping by sequencing (Table S1-S2). Each of these short genetic variants were associated with RBC cation phenotypes (*P* < 0.0001). The use of associated markers from the GWAS in the expanded animal cohort yielded strong evidence for an association (*P* < 10^^−99^) with all genetic variants. Therefore, an F-statistic was calculated to differentiate the levels of significance for each marker. Figure S5 shows F-values for each genetic variant tested and its genomic position on chromosome 18 of the reference domestic sheep genome. Linkage disequilibrium was calculated for all SNPs with an F statistic greater than 2000. Forty-seven variants showed strong LD with one another (all pairwise r^2^ ≥ 0.92), implicating one or a small number of very closely related haplotypes in the *MYADMF* region, including *MYADMF15A1*,* MYADMF1G1*,* MYADMF1H1*,* MYADMF3A1*, *MYADMF1B3*, and *MYADMF13C1.*

### Alphafold3 structural models and predicted protein interactions

**Fig. 2:**
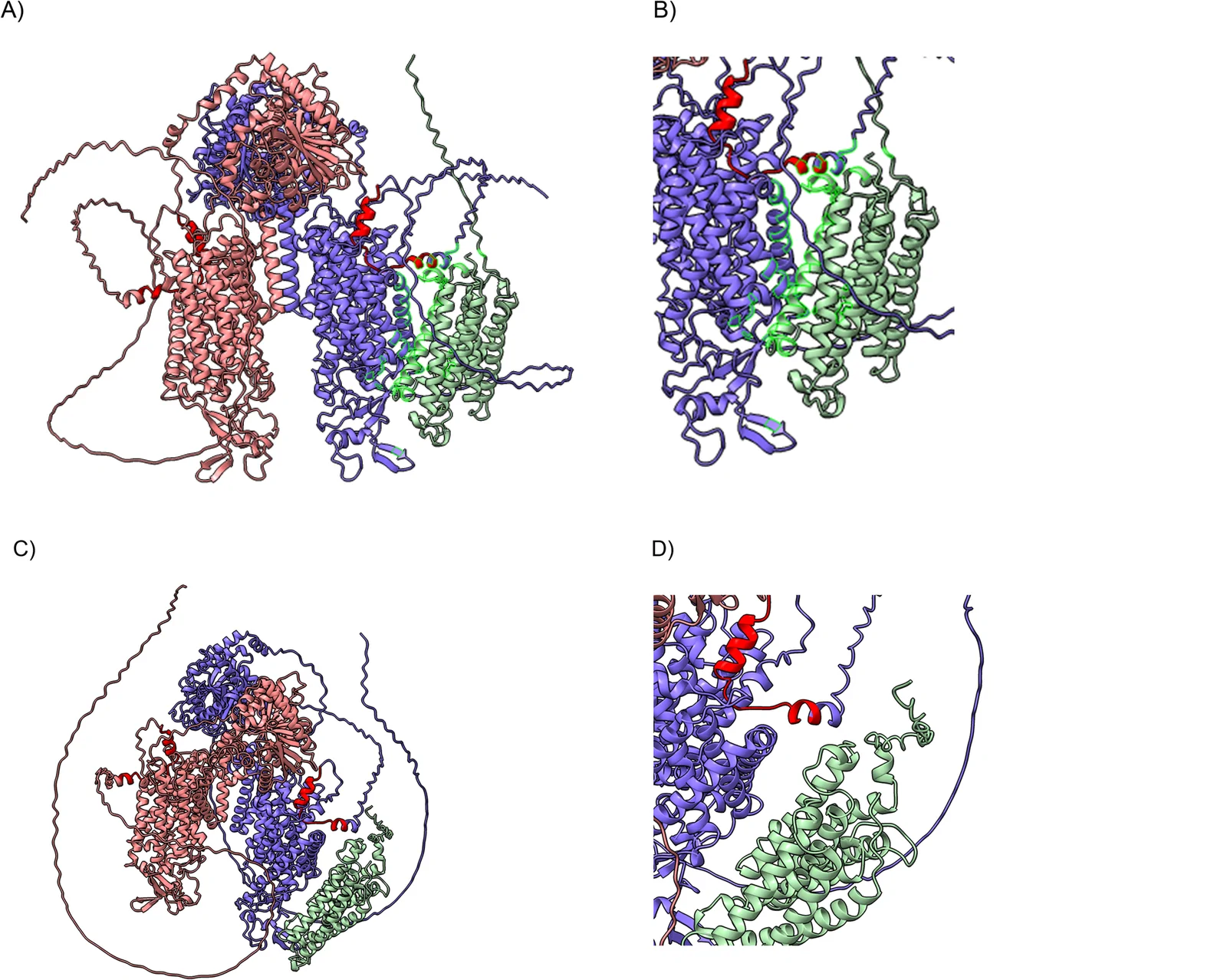
**A** Human MYADM interaction with KCC3A. MYADM is shown in green, and the two KCC3A subunits are shown in purple and pink. In all cases, the conserved N-terminal peptide of KCC3A is highlighted in red. **B** Human MYADM interaction with KCC3A, closer view. **C** Ovine MYADM interaction with KCC3A. **D** Ovine MYADM interaction with KCC3A, closer view

Alphafold3 generated a model of human MYADM interacting with the KCC3A dimer (iPTM = 0.61 and PTM = 0.67). Human MYADM interaction with KCC3A is shown in Fig. 2. Interacting residues for human MYADM and for KCC3A include those shown in Table S11. Alphafold3 also generated a model of human MYADM interacting with the KCC3B dimer (iPTM = 0.59 and PTM = 0.66).

Alphafold3 generated a model of sheep MYADM interacting with KCC3A dimer (iPTM = 0.60 and PTM = 0.66). The interaction between ovine MYADM and KCC3A is illustrated in Fig. 2. Interacting residues for sheep MYADM and for KCC3A include those shown in Table S12.

**Fig. 3:**
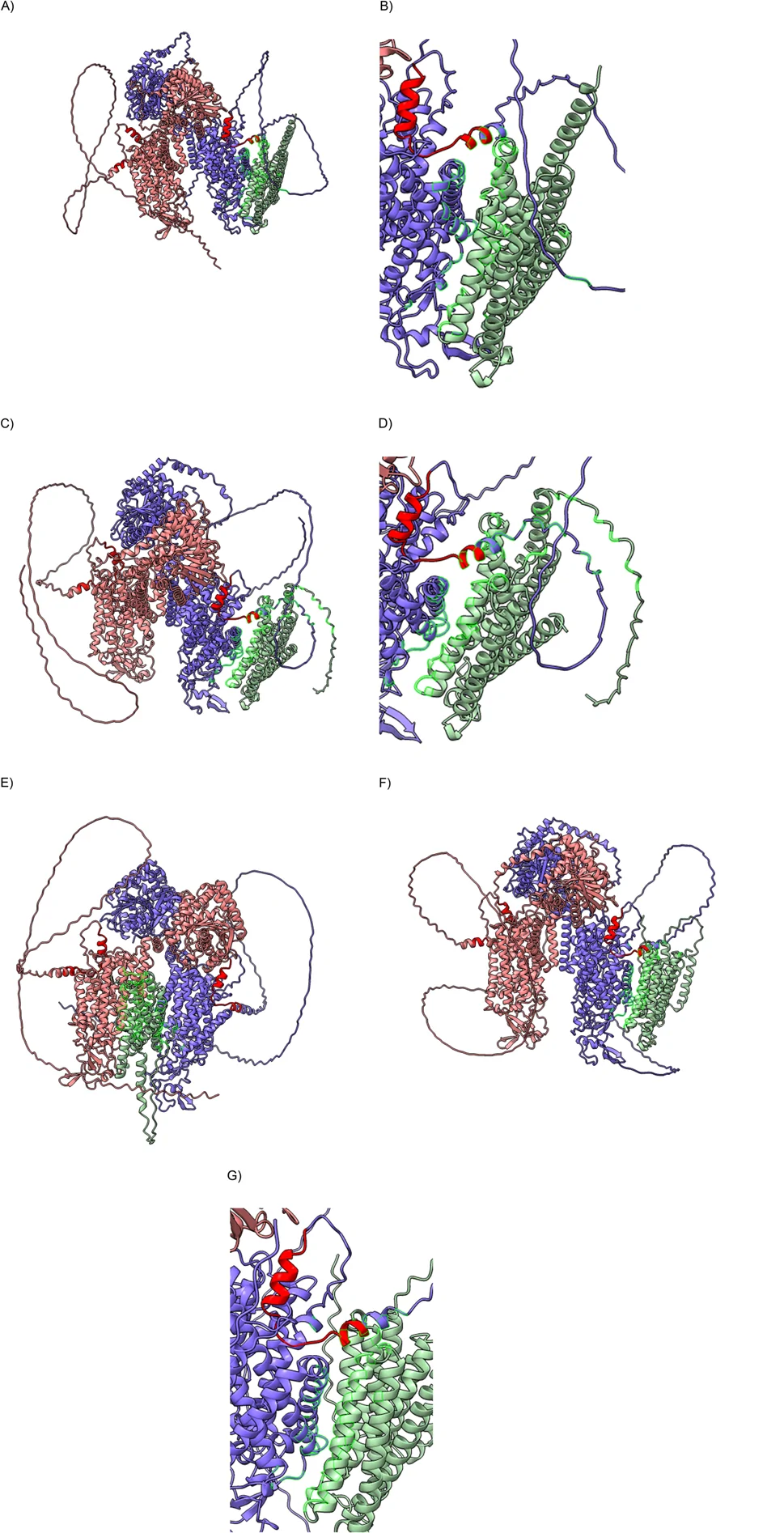
Interaction of ovine MYAMDF1G1 reference or alternate alleles with KCC3A, and MYADMF1B3 reference or alternate alleles with KCC3A. 3 **A**) The Texel v4 reference allele is shown in green with the two KCC3A subunits are shown in purple and pink. 3**B**) Closer view of 3 A. 3 **C**) A similar model of the MYADMF1G1 alternate (BAC-derived) allele. 3**D**) Closer view of 3 C. 3**E**) The MYADMF1B3 Texel v4 reference allele is shown in green with the two KCC3A subunits are shown in purple and pink. 3 **F**) A similar model of the MYADMF1B3 alternate (BAC-derived) allele. 3**G**) Closer view of 3G

Alphafold3 generated a model of sheep MYADMF1F1 interacting with KCC3A dimer (iPTM = 0.61 and PTM = 0.67). In addition, Alphafold3 generated a model of Texel OARv4 reference allele ovine MYADMF1G1 interacting with KCC3A dimer (iPTM = 0.61 and PTM = 0.67), and a model of alternate allele MYADMF1G1 interacting with KCC3A dimer (iPTM = 0.61 and PTM = 0.67). The overlap of the reference allele and the alternate allele MYADMF1G1 interaction with KCC3A is shown in Fig. 3. Interacting residues for sheep reference allele and alternate allele MYADMF1G1 and for KCC3A include those shown in Table S13.

Alphafold3 generated a model of Texel OARv4 reference allele ovine MYADMF1B3 interacting with KCC3A dimer (iPTM = 0.62 and PTM = 0.67), and a model of alternate allele MYADMF1B3 interacting with KCC3A dimer (iPTM = 0.59 and PTM = 0.65). The overlap of the reference allele and the alternate allele MYADMF1B3 interaction with KCC3A is shown in Fig. 3. Interacting residues for the sheep reference allele and alternate allele MYADMF1B3 and for KCC3A include those shown in Table S14.

Finally, Alphafold3 generated a model of Texel OARv4 reference allele ovine MYADMF15A1 interacting with KCC3A dimer (iPTM = 0.62 and PTM = 0.67), and a model of alternate allele MYADMF15A1 interacting with KCC3A dimer (iPTM = 0.61 and PTM = 0.67). The overlap of the reference allele and the alternate allele MYADMF15A1 interaction with KCC3A is shown in Figure S6.

## Discussion

For over 50 years, sheep LK and HK erythrocytes have served as physiologic models for cation transport, but the underlying gene(s) and mutation(s) responsible were unknown [39]. However, numerous studies from earlier decades defined many characteristics of the underlying mutation(s) [12, 13, 16, 23, 30, 95]. Here, we present data that demonstrate a *MYADMF* locus matches these criteria for the intraerythrocytic potassium concentration locus. These findings provide insight into the unique biology of artiodactyls and suggest that MARVEL domain proteins can act as upstream regulators of K-Cl cotransporters, which may explain the role of MYADM in human hypertension.

Previous studies indicated the intraerythrocytic potassium concentration locus is a single genetic locus that controls the LK/HK dimorphism [12]. Secondly, the alleles of this locus are common in many sheep breeds worldwide [23, 24]. While genome-wide association identified several SNPs that showed genome-wide association (*P* < 5 × 10^− 8^) with intracellular cation concentration phenotypes (Tables S3-S5), the single locus most highly associated with all cation phenotypes was located on ovine chromosome 18, directly overlapping a *MYADMF* gene expansion. Within this locus, four SNPs showed identical nominal *P*-values in the intraerythrocytic cation GWAS (1.1 × 10^− 63^). The four SNPs were located in a region containing *MYADMF1I1*,* MYADMF15A1*,* MYADMF1G1*,* MYADMF1H1*,* MYAMDF3A1*,* MYADMF1B3*, and *MYADMF13C1*. These four peak SNPs were in strong LD with previously identified divergent artiodactyl *MYADMF* repeat (*MYADMF1F1*) genotypes (D’=0.97, r^2^ = 0.87). This is the same locus that we previously identified as associated with red blood cell size and hemoglobin concentration traits in multiple breeds of domestic sheep [41].

Another characteristic of the intraerythrocytic potassium concentration locus identified in previous studies is that the LK and HK alleles are in high prevalence in the world’s sheep breeds [11, 23, 24]. Combined SNP data from over 3,000 sheep from 77 diverse breeds in the global ovine HapMap project and our prior work demonstrate wide global distribution with high allele frequencies in many breeds [41, 96].

Prior work on the LK/HK locus has also led to expectations for the intraerythrocytic potassium concentration locus to explain specific extreme cation concentrations in RBC [12, 97]. Our data show three distinct non-overlapping distributions of intracellular potassium concentration in sheep red cells segregating with alternate (HK) and reference alleles (LK) (Table 1) that are similar to reported potassium concentrations of 80–90 mM in HK cells and 20–30 mM in LK cells. Intracellular sodium levels varied inversely with intracellular potassium levels, with 30–40 mM in HK cells and 75–85 mM in LK red cells [23]. Although previous studies regarding sheep red blood cell potassium levels had measured total cation composition in whole blood [12, 23, 24], and this analysis used purified erythrocytes for intracellular concentration, the results are comparable because most of the potassium is derived from red blood cells in a whole blood analysis [98].

A further characteristic of the intraerythrocytic potassium concentration locus is nearly complete dominance of LK over HK alleles. Inheritance of the genetic variants within the *MYADMF* association peak demonstrate a nearly dominant mode of inheritance, with reference allele homozygotes (LK cells) showing markedly reduced intracellular potassium in red cells (Table 1). However, with the benefit of modern measurements and genotype determination, we observed that LK/HK heterozygotes have significantly different intracellular potassium concentrations compared to LK/LK homozygotes and HK/HK homozygotes, as determined using modern detection methods (Table 1). This finding is consistent with prior reports of the LK/HK locus, given the technological limitations of previous studies, where LK would appear dominant, and the much smaller difference between LK homozygotes and heterozygotes would have been undetectable or overlooked [30, 31, 97, 99].

To further characterize the *MYADMF* expanded region, we performed deep sequencing (to an average of>600X PacBio coverage) of a single BAC clone spanning beyond our locus on both sides from a non-reference homozygous sheep. It included ~ 295 kb of the alternate allele, which contained *MYADMF* genes, including *MYADMF11A1*,* MYADMF12A1*,* MYADMF2C1*,* MYADMF13A1*,* MYADMF1F4*,* MYADMF14A1*,* MYADMF13B1*,* MYADMF1B2*,* MYADMF1I1*,* MYADMF15A1*,* MYADMF1G1*,* MYADMF1H1*,* MYAMDF3A1*,* MYADMF1B3*, and *MYADMF13C1* in the sheep ARS-UI Ramb v.2.0 reference genome. Interestingly, one ORF appeared deleted in the alternate allele assembly, which was *MYADMF1F1*, an apparent full-length ORF with high sequence identity (> 70%) to the *MYADM* gene. The presence or absence of this gene has been highly associated with RBC phenotypes and lifetime ewe production traits [41]. Additionally, 6 ORFs were significantly lengthened (> 100 bps) in the alternate allele sequence compared to the reference allele, including *MYADMF13A1*, *MYADMF15A1*, *MYADMF1G1*, *MYADMFIH1*, *MYAMDF3A1*, and *MYADMF1B3.*

Since prior data indicated functional changes in potassium transport upon antibody binding [18, 100–102], initial fine mapping focused on variants encoding putative ORF changes in variants with an F statistic > 1200. Sixty-nine genetic variants within the locus ORFs resulting in missense or nonsense mutations were identified as candidate functional mutations and analyzed along with 60 synonymous variants in an expanded population. Importantly, the divergent artiodactyl *MYADM*-like repeat marker (*MYADMF1F1*), previously associated with RBC and production traits [41], had an F statistic greater than 800, clearly indicating that it is not the best marker for intracellular potassium in this region. In total, 47 variants were associated with RBC intracellular potassium and had F statistics greater than 2000 (Fig. S5). There was LD (r^2^ ≥ 0.92) between these 47 variants for all pairwise variant comparisons. These variants were found within six *MYADMF* ORFs, including: *MYADMF15A1*,* MYADMF1G1*,* MYADMF1H1*,* MYADMF3A1*, *MYADMF1B3*, and *MYADMF13C1.* One or more of these genes may be critical to the control of RBC intracellular cation concentration in domestic sheep. Since K-Cl co-transport has been shown to regulate activity of the NADPH oxidase and reactive oxygen species (ROS) generation in phagocytic cells [103], *MYADMF* genes may also regulate ROS-mediated pathogen killing. However, further work is needed to characterize these *MYADMF* genes in domestic sheep.

Another characteristic of the intraerythrocytic potassium concentration locus is similar segregating alleles for LK and HK in RBC in diverse ruminants. The locus associated with intraerythrocytic potassium concentration extends beyond domestic sheep to include many diverse ruminants including goat, cow, yak, water buffalo, wildebeest, and Speke’s gazelle, among others [33, 35–37, 104–107]. Comparable *MYADMF* gene expansions were identified in all artiodactyl reference genomes examined, including those of goat, cow, and pig. The goat (*Capra hircus*, v.1.2) and cow (*Bos taurus*, ARS-UCD1.2) reference genomes contain 33 and 52 *MYADMF* ORFs, respectively, with *MYADMF* expansions mapping to loci on chromosome 21. Like the expanded *MYADMF* gene expansion found in domestic sheep, many of these ORFs appear full-length when compared to the *MYADM* ORF, although many probable *MYADMF* pseudogenes (< 100 amino acids) also exist. No evidence was found of similar *MYADMF* expansions in more distantly related relatives of the artiodactyl lineage, including representative perissodactyls (horse, Przewalski’s horse, rhinoceros) and other mammals. These findings suggest that these loci arose after the artiodactyl split from other mammals but before the speciation of individual artiodactyl species. Additionally, these *MYADMF* loci have been evolving independently in each species. In particular, identification of a *MYADMF* locus in domestic pigs suggests searches for LK/HK dimorphism may be valuable in defining function for specific *MYADMF* alleles.

In addition, historical antibody recognition of LK/HK expression on the cell surface is consistent with mutations in *MYADMF* genes across species. Sheep anti-L antibody is recognized by goat and cow LK cells [108, 109]. Antibody binding altered potassium transport, suggesting the same locus is involved in these species [108, 109]. The MYADM protein is a MAL protein family member containing two MARVEL domains and eight total transmembrane domains, and MYADM has been shown to localize at the plasma membrane of developing myeloid cells [110–113]. The *MYADMF* ORFs identified at the sheep expanded *MYADMF* locus may have similar functions to the MYADM protein, or behave differently, as gene duplication is a common mechanism for neofunctionalization [114]. However, the high amino acid sequence identity (> 75%) and conservation of MARVEL transmembrane domains argues for the possible surface expression of these *MYADMF* members. Taken together, the characteristics of these genetic variants offer enticing candidates for further functional study into the possible mechanism of intracellular cation control in RBCs of domestic sheep.

The *MYADM* gene has been implicated in a number of studies involving hypertension in humans [45, 46], but little is known about the potential mechanism of the *MYADM* gene in the context of hypertension. The *MYADM* gene is highly expressed in neutrophils and other cells of the myeloid lineage, including erythrocytes [111, 115]. Prior studies have suggested that neutrophils can play important roles in blood pressure regulation [116]. The LK/HK sheep RBC were used to identify K-Cl cotransporter activity [5], and indeed, there was speculation that the LK/HK locus would be a simple structural variant in one of the K-Cl cotransporters [39]. These data suggest that *MYADMF* loci may be upstream regulators of K-Cl cotransporters, which have known involvement in blood pressure regulation [45, 46, 117].

The *MYADM* gene is a member of the MAL protein superfamily that all contain MARVEL domains [118]. MAL proteins play roles in apical sorting at the plasma membrane and have been shown to play an important role in stabilizing Na+-K+-2Cl- cotransporters (NKCC2) and regulating cation absorption in kidney cells [119]. The K-Cl cotransporter protein subfamily is represented by genes now known as *SLC12A3-SLC12A6* that encode proteins known as KCC1-KCC4, respectively [6, 20–22]. They are separate from but can interact with sodium-potassium ATPase [120]. The simplest hypothesis is an interaction of one (or more) of the *MYADMF* family members with one (or more) of the KCC molecules in mediating potassium efflux. However, to our knowledge, this paper is the first report of *MYADMF* genes contributing to intracellular cation concentration in any mammalian system and may offer insight into the functional significance of the MYADM protein in hypertension. This study reveals that the sheep K locus is an expanded *MYADMF* repeat, suggesting a role for MYADMF in regulating KCC upstream. The simplest upstream role would be direct interaction between MYADMF and KCC. However, the sheer number of *MYADMF* genes makes this a complex question to answer.

To begin more simply, we considered another long-standing mystery regarding the mechanism by which MYADM itself is involved in hypertension. It has been known for some time that MYADM was a top result in human hypertension studies [45–47]. There has been some work relating MYADM to hypertension [121], but much of this work has focused on MYADM as an effector of the miRNA, miR-182-3p [48, 49]. Building on our observation that MYADM family proteins regulate KCC proteins, we modeled the potential interaction of human MYADM with KCC3A (Fig. 2). The MYADM interaction stabilizes the N-terminal peptide in the ion-binding groove (Fig. 2). This interaction configuration is similar in structure for both human MYADM with KCC3B (data not shown) and sheep MYADM interactions with KCC3A (Fig. 2). It is known that KCC3 knockout induces hypertension [122, 123], by stabilizing the KCC3 inhibitory N-terminal domain, our model explains how MYADM might influence hypertension. Both MYADM and KCC3 are expressed in vascular smooth muscle and the kidney, among other tissues, where KCC3 could exert effects on systemic blood pressure [124–126]. Thus, we propose that one key mechanism for MYADM involvement in hypertension is in interaction with KCC3. This mechanism enables tissue-specific honing through tissue-specific expression differences, both in terms of presence or absence and relative abundance. While the structure iPTM scores were slightly lower, the overall sequence conservation, the conservation of the N-terminal peptide mechanism, and the specific sequence conservation of the N-terminal peptide and interface sites [127–129] could enable similar MYADM interactions with other KCC transporters. Indeed, models for KCC1, KCC2, and KCC4 with MYADM exhibit similar interactions, albeit with slightly lower iPTM scores (0.57–0.59, except for KCC4, where iPTM = 0.54).

We then returned to the more complex issue of MYADMF protein interaction with KCC3A in relation to sheep erythrocyte potassium concentration. As a first test, we used Alphafold3 [52] to generate structures of MYADMF proteins in combination with ovine KCC3A dimers since KCCs function as dimers and KCC3A is the predominantly expressed isoform in erythrocytes [91, 130]. We focused initially on MYADMF1F1 since it was the only gene absent on the alternate allele but present on the reference allele [41]. However, the strong dominant inheritance of the alternate allele would be difficult to explain for MYADMF1F1, which is absent in the alternate allele. Indeed, MYADMF1F1 is a full-length repeat protein that is predicted to bind KCC3A in the transmembrane region near the cell surface. The binding is predicted to occur between the KCC3A proteins in the dimer, which may have a minimal impact on KCl cotransport.

An examination of the LK alternate allele from the BAC sequence included models for all genes in the critical region. We believe the dominance of the LK allele for erythrocyte potassium strongly argues for one or more factors present on the LK/alternate allele but absent on the reference allele. Based on the structures we generated, we identify three mechanisms that could be applicable, and they are not mutually exclusive. (1) One or more MYADMF (MYADMF1G1 and/or MYADMF1B3, possibly others) interacts with the exterior of KCC3A and can block binding of the N-terminal peptide. (2) One or more MYADMF, such as MYADMF15A1, bind KCC and, through allosteric interactions, influence N-terminal peptide binding. (3) One or more MYADMF bind KCC and influence potassium cotransport through mechanisms other than N-terminal peptide function, such as through allosteric changes affecting the transport mechanism itself.

The first hypothesis is based on large differences in predicted KCC3A function, particularly in the N-terminal peptide observed for MYADMF1G1 and MYADMF1B3. For both MYADMF1G1 and MYADMF1B3, the alternate allele is predicted to bind KCC3A in such a way as to interface with the N-terminal peptide and possibly interfere with its function (Fig. 3). Conversely, neither reference allele (MYADMF1G1 or MYADMF1B3) has any predicted interaction with the N-terminal regulatory peptide (Fig. 3). It is known that the N-terminal regulatory loop plays an inhibitory role in potassium chloride cotransport, and mutants lacking this N-terminal loop are constitutively active [127–129]. MYADMF1G1 or MYADMF1B3 binding to stabilize the N-terminal loop outside the ion binding groove would have a similar effect and would explain the known high transport activity from low K erythrocytes [122]. The structure shows stress on the N-terminal peptide from MYADMF1G1 or MYADMF1B3 binding in the form of lower confidence of correct folding, particularly for the interface residues, but does not show the N-terminal peptide dislodged. However, AlphaFold has a tendency to model only one conformation among many (probably due to biases in training input data, where some conformations have been more common and perhaps more easily observed experimentally) [131]. The N-terminal peptide in place is likely the preferred configuration based on known structures used for training Alphafold3 [132]. This explanation is certainly within the realm of experience for AlphaFold models [133, 134]. We note that AlphaFold3 is generally helpful at predicting mutational effects [135–137], but there is room to improve mutational effect prediction. In particular, AlphaFold3 has limitations in predicting multiple conformational states of the same protein. Additionally, for any unstructured domains, it is challenging for AlphaFold3 to predict conformation with confidence [138].

*MYADMF1G1* is quite divergent between the Texel v4 reference and the BAC allele, where the BAC allele has 28 additional N-terminal residues, plus 21 additional amino acid changes, including 6 charged substitutions and one in-frame residue deletion. MYADMF1B3 is even more divergent between haplotypes with 54 additional N-terminal residues compared to the Texel v4 reference allele, plus 34 other amino acid changes (including 8 charged substitutions). The amino acid identity at the C-terminal end after F229 diverges sharply, with the Texel v4 reference possessing 56 serine-rich residues at the C-terminal end, while the BAC allele possesses 90 very divergent residues.

A second model of MYADMF regulation of KCC based on the N-terminal peptide is exemplified by MYADMF15A1. In this case, allosteric interactions from the binding of the alternate allele but not the Texel v4 allele change the length of the alpha helix in the N-terminal peptide itself (Figure S6). While the apparent changes are subtle, they could alter the regulatory function of the N-terminal peptide. For example, the allosteric changes could apply pressure, making it less likely that the N-terminal peptide fits the binding groove once it is outside of the cell.

Finally, there are potential mechanisms that do not involve the N-terminal peptide. For example, KCCs are also regulated by phosphorylation, where alanine substitution of highly conserved sites T991 and T1048 has been shown to create constitutively active cotransporters [139]. These sites can be affected by interactions with MYADMF, or allosteric interactions may alter the functional implications of their phosphorylation or dephosphorylation. The expression pattern of MYADMF genes is not well understood, so the presence of these genes in other tissues, such as those other than erythrocytes, where constitutive KCC3 activation might occur, remains unclear. Nonetheless, MYADMF1G1 might play a key role in the low K phenotype.

The most economically important phenotype association with the *MYADMF* locus was made in our prior work [41]. The fact that the association was with a complex, cumulative trait - ewe lifetime weight of lambs weaned - may explain why other, smaller studies with fewer years of phenotypic records had not found it in the past. It is not clear at present what the mechanism for the difference in lifetime weight of lambs weaned might be, despite the clear hypotheses for involvement in blood potassium presented here. In addition, prior work in sheep had concluded that fleece weight might be associated with the LK/HK locus [140]. However, this would need to be validated with larger animal datasets. Additionally, the *MYADMF* locus identified impacts intracellular potassium levels, which may explain QTLs contributing to altered hematocrit and milk yield found at the same genetic locus on chromosome 18 [141, 142].

## Conclusions

Overall, we have identified the *MYADMF* expanded repeat locus as the genetic determinant of the sheep low versus high K erythrocyte phenotype. It has long been known that the physiology of sheep LK and HK erythrocytes is driven by KCC proteins, highlighting MYADMF’s upstream control of KCC function.

While structural models offer only hypotheses for experimental testing, it is intriguing that Alphafold3 models show conservation of proposed mechanisms across species. Alphafold3 models not only show potential for direct interaction between MYADMF proteins and KCC3A, but allele differences in MYADMF1G1 or MYADMF1B3 might play a key role in accounting for the HK versus LK phenotype through interaction with the N-terminal peptide. Alternatively, MYADMF15A1 binding might influence N-terminal peptide regulation through allosteric effects. Furthermore, both human and sheep MYADM appear to interact with KCC3A (and other KCCs) by stabilizing the N-terminal peptide in the inhibitory position, which may explain how MYADM is involved in hypertension. Additional work will be required to assess the testable hypotheses for functional involvement provided herein and to characterize the interaction of MYADMF and MYADMF proteins with KCC N-terminal peptides. Further work is also needed to characterize the *MYADMF* clusters in domestic sheep and other livestock species, as well as to investigate the possible functional significance of these clusters to various phenotypes related to potassium transport in myeloid cells.

An important direction for future work is gene expression of the *MYADMF* loci. Such expression data are more difficult to collect in erythrocytes, but erythrocyte precursors and other cell types can help leverage gene expression and illuminate its contributions to phenotype. Additionally, while this study was clearly well powered to detect a single gene trait like LK/HK, it was less well powered to detect the other loci associated with various traits. Nonetheless, several additional associated loci were identified and further work may help clarify specific mutations and their roles.

## Supplementary Information

Supplementary Material 1. Figure S1. Manhattan plot of erythrocyte sodium concentration. Figure S2. Manhattan plot of erythrocyte sodium/potassium ratio. Figure S3. MYADM family expanded tandem repeat locus comparative map. Figure S4. Phylogenetic tree of expanded MYADM family genes. Figure S5. F curves by cation phenotype. Figure S6. Ovine MYADMF15A1 interaction with KCC3A. S6A) The Texel v4 reference allele is shown in green with the two KCC3A subunits are shown in purple and pink. S6B) A similar model of the MYADMF15A1alternate (BAC-derived) allele interacting with KCC3A.

Supplementary Material 2. Table S1. PCR primers. Table S2. Taqman primers and probes. Table S3. SNP associated with erythrocyte potassium. Table S4. SNP associated with erythrocyte sodium. Table S5. SNP associated with erythrocyte sodium/potassium ratio. Table S6-9. MYADMF loci, positions, and nomenclature from sheep (S6), cow (S7), goat (S8), and pig (S9). Table S10: Genetic variants within MYADMF ORFs. Table S11. Human MYADM and KCC3A predicted interacting residues. Table S12. Ovine MYADM and KCC3A predicted interacting residues. Table S13. Predicted interacting residues for ovine reference or alternate allele MYADMF1G1 and KCC3A. Table S11. Predicted interacting residues for ovine reference or alternate MYADMF1B3 and KCC3A.

## Data Availability

The datasets supporting the conclusions of this article are available in the GenBank repository, accession PX438062 (https://www.ncbi.nlm.nih.gov/nuccore/PX438062.1/), and in the Open Science Framework repository, DOI 10.17605/OSF.IO/9CMY7 (https://osf.io/9cmy7/overview). Additional datasets supporting the conclusions of this article are included within the article and its additional files.

## Abbreviations

BAC: Bacterial artificial chromosome
BAM: Binary alignment/map (file format)
BLAST: Basic local alignment search tool
GWAS: Genome-wide association study
HGAP: Hierarchical Genome Assembly Process
HK: High potassium concentration red blood cell
iPTM: Interface predicted template modeling score
KCC: Potassium chloride cotransporter
LD: Linkage disequilibrium
LK: Low potassium concentration red blood cell
MARVEL: Membrane-associating lipid-vesicle-exchanging-related lipoprotein
MCHC: Mean corpuscular hemoglobin concentration
MCV: Mean corpuscular volume
MEGA: Molecular evolutionary genetics analysis
miRNA: MicroRNA
MUSCLE: Multiple sequence comparison by log-expectation
MYADM: Myeloid-associated differentiation marker
MYADMF: Myeloid-associated differentiation marker family
ORF: Open reading frame
PCR: Polymerase chain reaction
PTM: Predicted template modeling score
QTL: Quantitative trait locus
RBC: Red blood cell
ROS: Reactive oxygen species
SMRT: Single molecule, real-time
SNP: Single nucleotide polymorphism
