# Supplementary material for: Sheep erythrocyte potassium locus highlights MYADM family regulation of electroneutral potassium chloride cotransporters (KCC)
Source: BMC Genomics. 2026 Jun 16;27:710. doi: 10.1186/s12864-026-13029-7 (PMC13508224; doi:10.1186/s12864-026-13029-7)

Supplementary Figures

Figure S1. Manhattan plot of erythrocyte sodium concentration

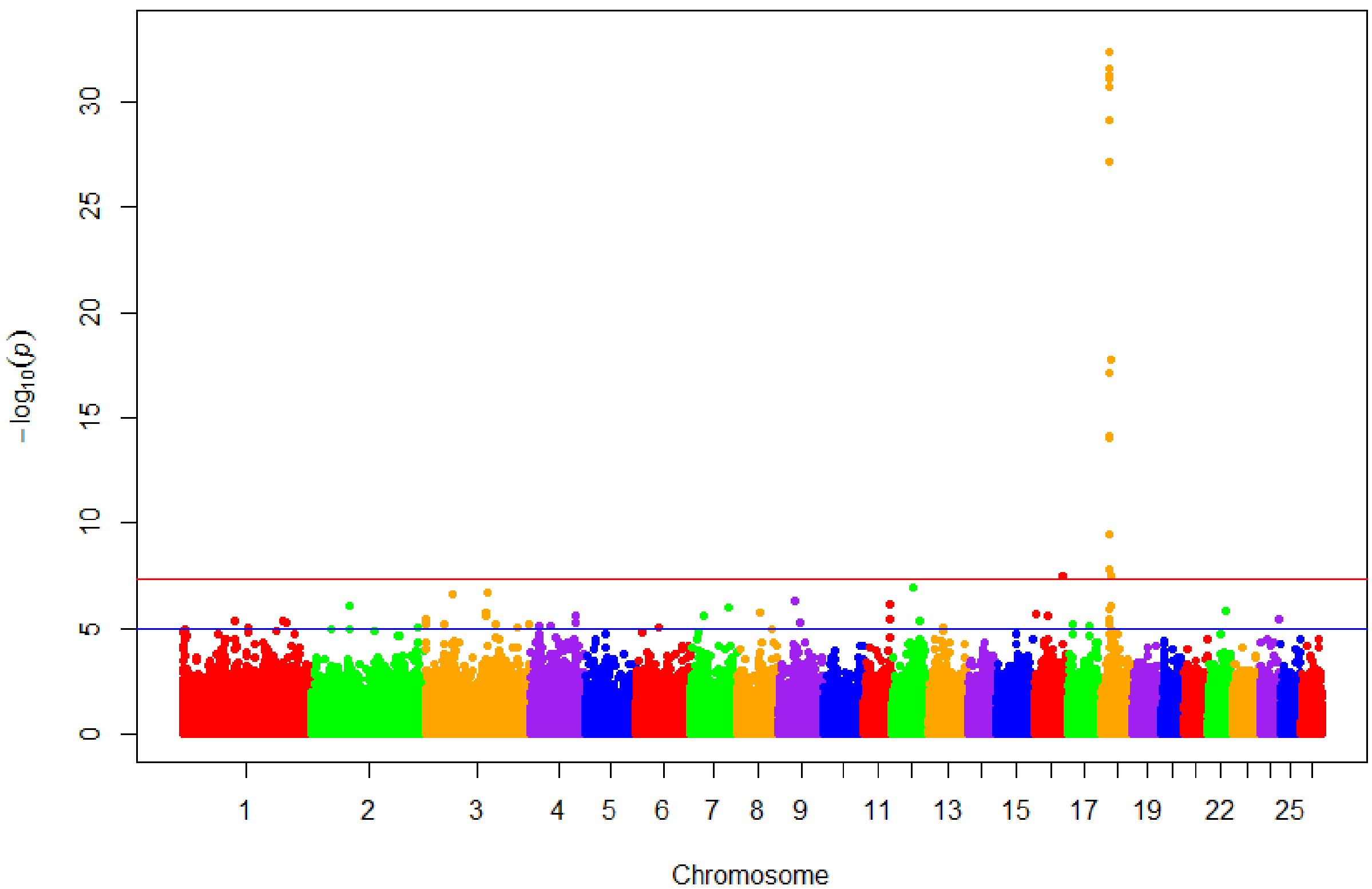

The genome-wide suggestive threshold (red line) was 5 x 10^{-8}, and the genome-wide suggestive threshold (blue line) was 1 x 10^{-5}.

Figure S2. Manhattan plot of erythrocyte sodium/potassium ratio

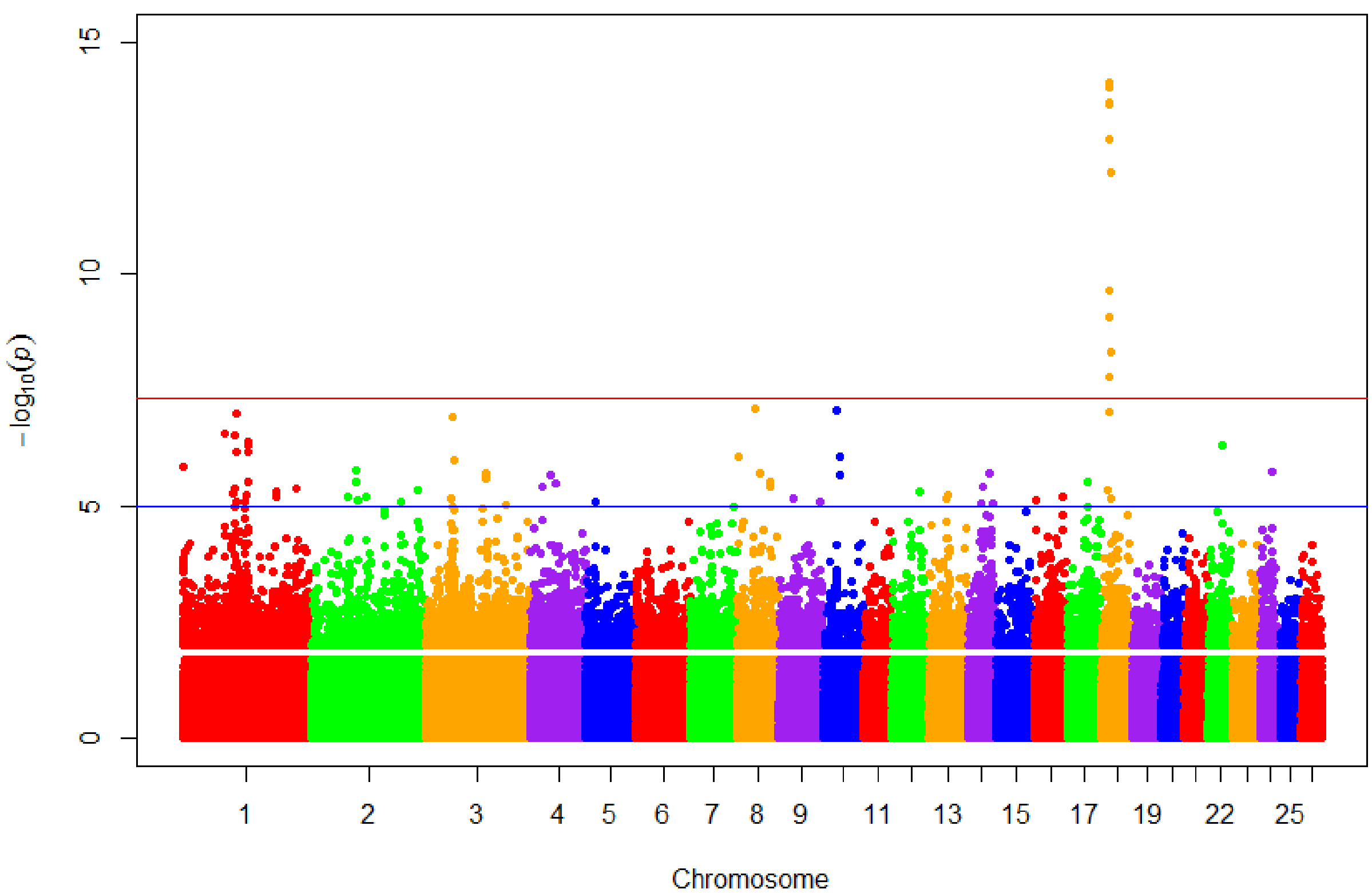

The genome-wide suggestive threshold (red line) was 5 x 10^{-8}, and the genome-wide suggestive threshold (blue line) was 1 x 10^{-5}.

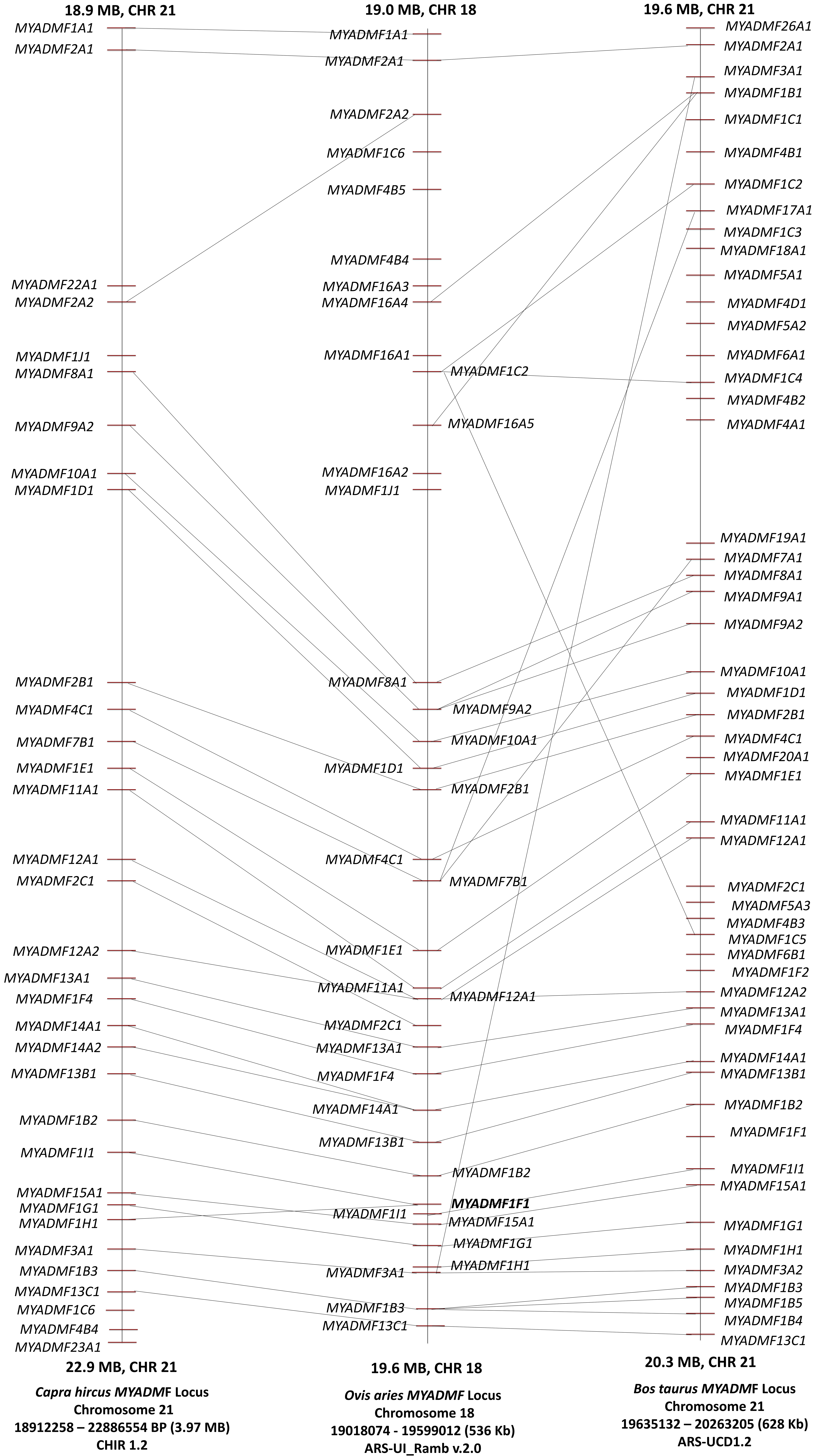
18.9 MB, CHR 21
MYADMF1A1
MYADMF2A1
MYADMF22A1
MYADMF2A2
MYADMF1J1
MYADMF8A1
MYADMF9A2
MYADMF10A1
MYADMF1D1
MYADMF2B1
MYADMF4C1
MYADMF7B1
MYADMF1E1
MYADMF11A1
MYADMF12A1
MYADMF2C1
MYADMF12A2
MYADMF13A1
MYADMF1F4
MYADMF14A1
MYADMF14A2
MYADMF13B1
MYADMF1B2
MYADMF1I1
MYADMF15A1
MYADMF1G1
MYADMF1H1
MYADMF3A1
MYADMF1B3
MYADMF13C1
MYADMF1C6
MYADMF4B4
MYADMF23A1
22.9 MB, CHR 21
Capra hircus MYADMF Locus
Chromosome 21
18912258 – 22886554 BP (3.97 MB)
CHIR 1.2
19.0 MB, CHR 18
MYADMF1A1
MYADMF2A1
MYADMF2A2
MYADMF1C6
MYADMF4B5
MYADMF4B4
MYADMF16A3
MYADMF16A4
MYADMF16A1
MYADMF1C2
MYADMF16A5
MYADMF16A2
MYADMF1J1
MYADMF8A1
MYADMF9A2
MYADMF10A1
MYADMF1D1
MYADMF2B1
MYADMF4C1
MYADMF7B1
MYADMF1E1
MYADMF11A1
MYADMF12A1
MYADMF2C1
MYADMF13A1
MYADMF1F4
MYADMF14A1
MYADMF13B1
MYADMF1B2
MYADMF1F1
MYADMF1I1
MYADMF15A1
MYADMF1G1
MYADMF1H1
MYADMF3A1
MYADMF1B3
MYADMF13C1
19.6 MB, CHR 18
Ovis aries MYADMF Locus
Chromosome 18
19018074 - 19599012 (536 Kb)
ARS-UI_Ramb v.2.0
19.6 MB, CHR 21
MYADMF26A1
MYADMF2A1
MYADMF3A1
MYADMF1B1
MYADMF1C1
MYADMF4B1
MYADMF1C2
MYADMF17A1
MYADMF1C3
MYADMF18A1
MYADMF5A1
MYADMF4D1
MYADMF5A2
MYADMF6A1
MYADMF1C4
MYADMF4B2
MYADMF4A1
MYADMF19A1
MYADMF7A1
MYADMF8A1
MYADMF9A1
MYADMF9A2
MYADMF10A1
MYADMF1D1
MYADMF2B1
MYADMF4C1
MYADMF20A1
MYADMF1E1
MYADMF11A1
MYADMF12A1
MYADMF2C1
MYADMF5A3
MYADMF4B3
MYADMF1C5
MYADMF6B1
MYADMF1F2
MYADMF12A2
MYADMF13A1
MYADMF1F4
MYADMF14A1
MYADMF13B1
MYADMF1B2
MYADMF1F1
MYADMF1I1
MYADMF15A1
MYADMF1G1
MYADMF1H1
MYADMF3A2
MYADMF1B3
MYADMF1B5
MYADMF1B4
MYADMF13C1
20.3 MB, CHR 21
Bos taurus MYADMF Locus
Chromosome 21
19635132 – 20263205 (628 Kb)
ARS-UCD1.2

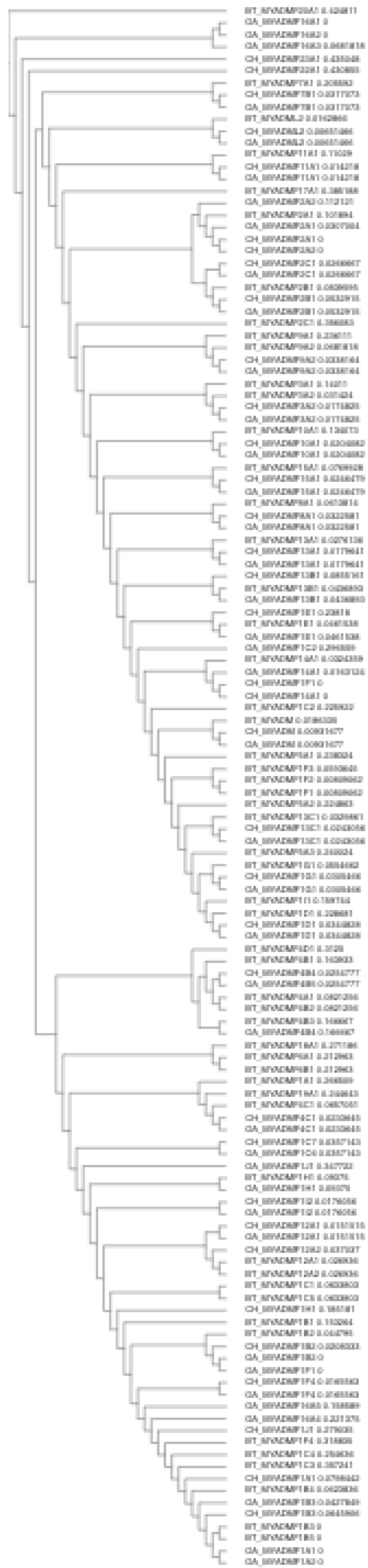

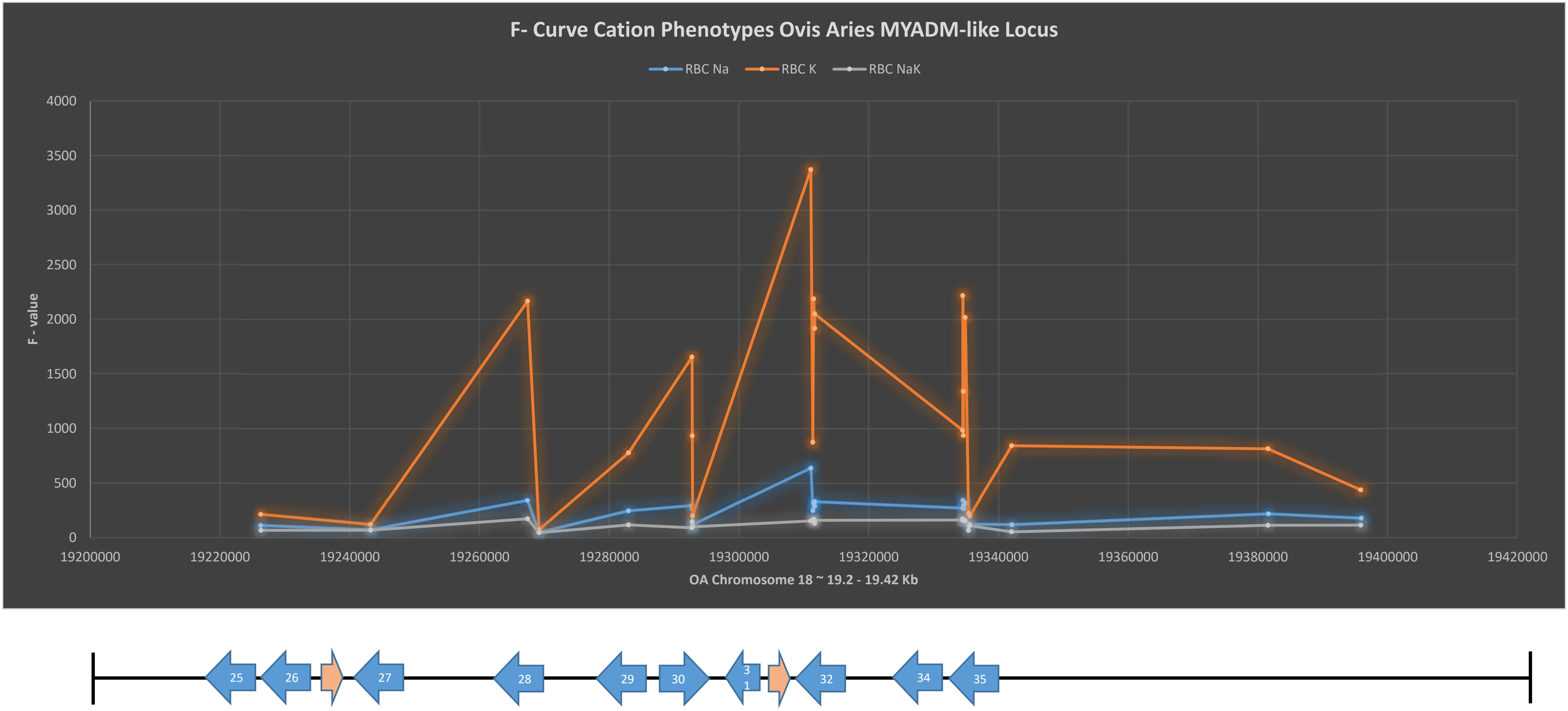

F- Curve Cation Phenotypes Ovis Aries MYADM-like Locus
RBC Na
RBC K
RBC NaK
4000
3500
3000
2500
2000
1500
1000
500
0
F - value
19200000
19220000
19240000
19260000
19280000
19300000
19320000
19340000
19360000
19380000
19400000
19420000
OA Chromosome 18 ~ 19.2 - 19.42 Kb
25
26
27
28
29
30
3
1
32
34
35

Figure S6. Ovine MYADMF15A1 interaction with KCC3A.

Figure S6A) The Texel v4 reference allele is shown in green with the two KCC3A subunits are shown in purple and pink.

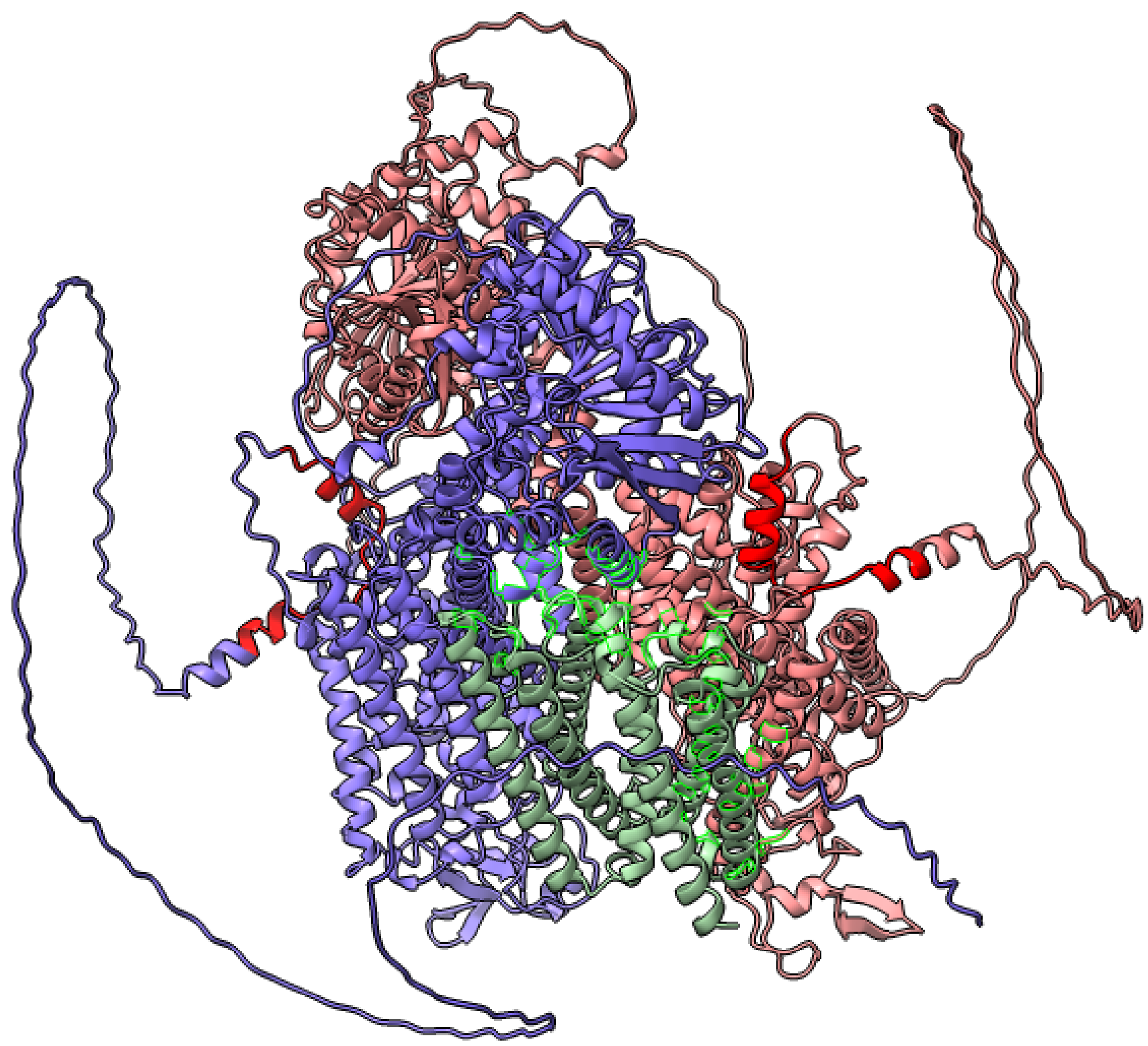

S6B) A similar model of the MYADMF15A1alternate (BAC-derived) allele interacting with KCC3A.

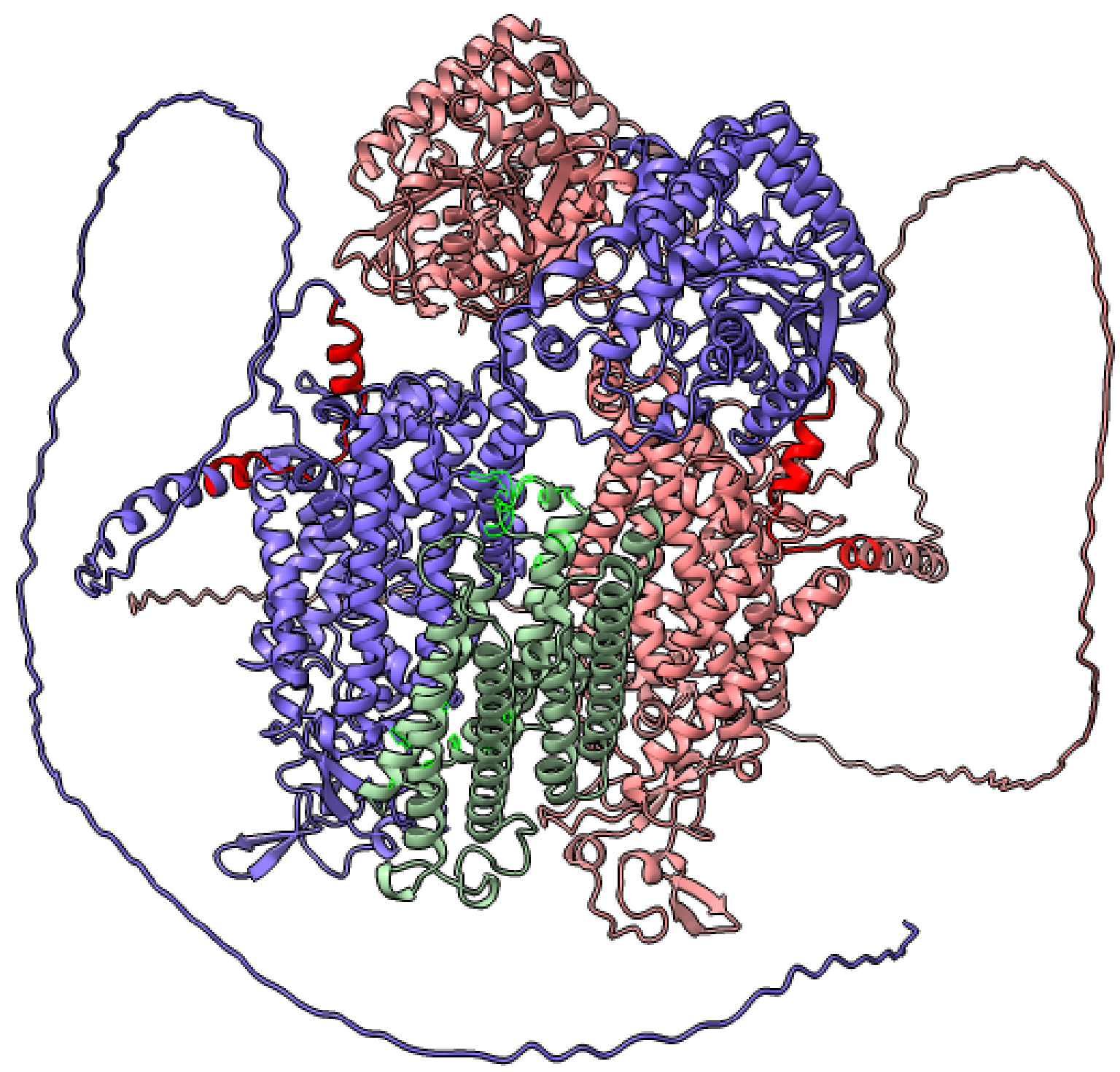

Supplement: Supplementary file 1 [file 12864_2026_13029_MOESM1_ESM.pdf]
