# Supplementary material for: Sheep erythrocyte potassium locus highlights MYADM family regulation of electroneutral potassium chloride cotransporters (KCC)
Source: BMC Genomics. 2026 Jun 16;27:710. doi: 10.1186/s12864-026-13029-7 (PMC13508224; doi:10.1186/s12864-026-13029-7)
Supplement: Supplementary file 2 [file 12864_2026_13029_MOESM2_ESM.zip › Supplementary Tables S1-S5 and S10-S13.docx]

Table S1: PCR, Sequencing, and Allele Specific Primer Sets used for Genotyping of NaK Expanded Population

| ***MYADMF* ORF** | **Primer** | **v.3.1 Start** | **v.3.1 Stop** | **Primer Length** | **Sequence (5’-3’)** |
| --- | --- | --- | --- | --- | --- |
| *MYADMF5B1* | Forward PCR | 19268273 | 19268293 | 21 | TCCGGTGAGAAGAGGCAATCA |
| *MYADMF5B1* | Reverse PCR | 19269249 | 19269269 | 21 | CCTGCTGTCAGTGATCCACAT |
| *MYADMF5B1* | Forward Seq 1 | 19268330 | 19268347 | 18 | AGATCACCAGGTCAACCA |
| *MYADMF5B1* | Forward Seq 2 | 19268695 | 19268714 | 20 | AGGATGCCACAGGCCACAGT |
| *MYADMF5B1* | Reverse Seq 1 | 19269243 | 19269263 | 21 | GTCAGTGATCCACATGACCAC |
| *MYADMF5B1* | Reverse Seq 2 | 19268756 | 19268777 | 22 | ACCTGTGTCAACACTAGTGAGC |
| *MYADMF1A5* | Ref. Allele | 19271842 | 19271864 | 23 | TGCAAGACTGGTTCACCCCAGAT |
| *MYADMF1A5* | Ref. Allele | 19272149 | 19272172 | 24 | CCGGCTTTATGTCAGTGATACGTG |
| *MYADMF1A5* | Alt. Allele | NA | NA | 23 | TAGAAGTACTCCTACCTGAACGA |
| *MYADMF1A5* | Alt. Allele | NA | NA | 23 | CCAAACAAATCCCTGAGCCATTT |
| *MYADMF1H1* | Forward PCR | 19323793 | 19323813 | 21 | GGTTACTCAGGAAGATCCTGC |
| *MYADMF1H1* | Reverse PCR | 19324818 | 19324839 | 22 | AGGAAGAGGGCATCAGAACTCT |
| *MYADMF1H1* | Forward Seq 1 | 19323797 | 19323817 | 21 | ACTCAGGAAGATCCTGCCACC |
| *MYADMF1H1* | Forward Seq 2 | 19324272 | 19324293 | 22 | GCGCAGTAAGATCTACAAGACC |
| *MYADMF1H1* | Reverse Seq 1 | 19324814 | 19324835 | 22 | AGAGGGCATCAGAACTCTGTAA |
| *MYADMF1H1* | Reverse Seq 2 | 19324353 | 19324374 | 22 | GCTGATGAAGGCGAAGATGATG |
| *MYADMF11C1* | Forward PCR | 19353380 | 19353401 | 22 | GTGGTCAGCACATAGGAGATGA |
| *MYADMF11C1* | Forward Seq | 19353582 | 19353603 | 22 | GAGCCAGGAAGAGAACATCAGA |
| *MYADMF11C1* | Reverse PCR | 19354584 | 19354605 | 22 | ATGACCTAGGCCTGCACAACCA |
| *MYADMF11C1* | Reverse Seq | 19354523 | 19354544 | 22 | TGACCATCGTCATGGGCTACTT |

Table S2: Taqman assay primers and probes used for genotyping in NaK Expanded Population

| **Number** | **Region** | **BAC Position** | **SNP Call** | **Primer** | **Sequence** |
| --- | --- | --- | --- | --- | --- |
| 1 | Outer Peak | 158104 | M | Forward Primer | GGGCCTTCTCTAAGGTCAAAGTTTA |
| 1 | Outer Peak | 158104 | M | Reverse Primer | GCGTTTAAATGCATGGCTTTGG |
| 1 | Outer Peak | 158104 | M | Vic Probe | CATTGTTGTAGGAGGTTAC |
| 1 | Outer Peak | 158104 | M | Fam Probe | CATTGTTGTATGAGGTTAC |
| 2 | *MYADMF12A1* | 175021 | R | Forward Primer | CCAATAGGTTGATGAATGTCAGGAT |
| 2 | *MYADMF12A1* | 175021 | R | Reverse Primer | TCACCTACACCATTTGCACCAT |
| 2 | *MYADMF12A1* | 175021 | R | Vic Probe | CACAGCCAGTTCC |
| 2 | *MYADMF12A1* | 175021 | R | Fam Probe | CAGCCAGTCCCCGG |
| 3 | Peak SNP | 199195 | R | Forward Primer | GTTGTCTTCAAGAAACTGCAGCTTT |
| 3 | Peak SNP | 199195 | R | Reverse Primer | TTTTAAAGCTAGCGAAGGGCAGAT |
| 3 | Peak SNP | 199195 | R | Vic Probe | ATTCCCCAGAAAGTAG |
| 3 | Peak SNP | 199195 | R | Fam Probe | ATTCCCCAGGAAGTAG |
| 4 | *MYADMF1I1* | 224546 | Y | Forward Primer | TGGACATATGCAGTGGAGTAGATGA |
| 4 | *MYADMF1I1* | 224546 | Y | Reverse Primer | CCTGGTTTTCTTTCTACTGGTACGA |
| 4 | *MYADMF1I1* | 224546 | Y | Vic Probe | TAGCGGGCTTAGATAA |
| 4 | *MYADMF1I1* | 224546 | Y | Fam Probe | TAGCGGGCTTAGGTAA |
| 5 | *MYADMF1I1* | 224585 | Y | Forward Primer | GGTGGCATAGCGGGCTTAG |
| 5 | *MYADMF1I1* | 224585 | Y | Reverse Primer | CCTCATCATCTCCATTGTTGAGTT |
| 5 | *MYADMF1I1* | 224585 | Y | Vic Probe | AACCAGGACAGGAGA |
| 5 | *MYADMF1I1* | 224585 | Y | Fam Probe | AAAGAAAACCAGGACGGG |
| 6 | *MYADMF1I1* | 224633 | R | Forward Primer | TCACAGAACTCAACAATGGAGATGA |
| 6 | *MYADMF1I1* | 224633 | R | Reverse Primer | GGGCATCTGGAGAGGAGTCA |
| 6 | *MYADMF1I1* | 224633 | R | Vic Probe | TCACAGCAAAACAG |
| 6 | *MYADMF1I1* | 224633 | R | Fam Probe | TCACAGCAAAATAG |
| 7 | *MYADMF1I1* | 224799 | R | Forward Primer | TGCCCCATTCATCCTTTTCT |
| 7 | *MYADMF1I1* | 224799 | R | Reverse Primer | GACTGTGACCTGCCAGATTTCA |
| 7 | *MYADMF1I1* | 224799 | R | Vic Probe | CTGGTGGGCATGGT |
| 7 | *MYADMF1I1* | 224799 | R | Fam Probe | CTGGTGGGCACGGT |
| 8 | ISG20 Exon 3 | 231466 | R | Forward Primer | CCAGGGACCCAGGACAAAGT |
| 8 | ISG20 Exon 3 | 231466 | R | Reverse Primer | AGATGTTTCTAGAGTGCTCCTTGCT |
| 8 | ISG20 Exon 3 | 231466 | R | Vic Probe | CTGGATTCGCTGAAG |
| 8 | ISG20 Exon 3 | 231466 | R | Fam Probe | CTGGATTCACTGAAGAG |
| 9 | ISG20 Exon 3 | 231490 | R | Forward Primer | GCACCAGGGACCCAGGAC |
| 9 | ISG20 Exon 3 | 231490 | R | Reverse Primer | GACTTGGCCTCAGACCCAGAG |
| 9 | ISG20 Exon 3 | 231490 | R | Vic Probe | CTCCTTGCTCAGTACAT |
| 9 | ISG20 Exon 3 | 231490 | R | Fam Probe | CTCCTTGCTCAGCACA |
| 10 | *MYADMF11C1* | 243168 | K | Forward Primer | CAGAGAGCAGGGTCAGCCC |
| 10 | *MYADMF11C1* | 243168 | K | Reverse Primer | TGGTGCGTGGCTGTGTACTC |
| 10 | *MYADMF11C1* | 243168 | K | Vic Probe | AGGGTCACTGCTGAC |
| 10 | *MYADMF11C1* | 243168 | K | Fam Probe | AGGAGGGTCACGGC |
| 11 | *MYADMF11C1* | 243266 | R | Forward Primer | CGGCTGGTGCACGTACAG |
| 11 | *MYADMF11C1* | 243266 | R | Reverse Primer | CCCGGTCTGGAGAGTTCTCC |
| 11 | *MYADMF11C1* | 243266 | R | Vic Probe | ATGACACAGGCCA |
| 11 | *MYADMF11C1* | 243266 | R | Fam Probe | ATGATGCAGGCCAC |
| 12 | *MYADMF11C1* | 243296 | Y | Forward Primer | GCTGATGAAGGCGAAGATGA |
| 12 | *MYADMF11C1* | 243296 | Y | Reverse Primer | CCTTGCATCTGTGGCTTATGC |
| 12 | *MYADMF11C1* | 243296 | Y | Vic Probe | TCCAGCCTGTGAAGCA |
| 12 | *MYADMF11C1* | 243296 | Y | Fam Probe | TCCAGCCTGTGAAACA |
| 13 | *MYADMF11C1* | 243467 | R | Forward Primer | GGCAGCAACTGGATGCAG |
| 13 | *MYADMF11C1* | 243467 | R | Reverse Primer | CACCTTCTTATGCCTCTCAGGCT |
| 13 | *MYADMF11C1* | 243467 | R | Vic Probe | GGTGGCAAAGACGA |
| 13 | *MYADMF11C1* | 243467 | R | Fam Probe | GGTGGCAAAGATGA |
| 14 | *MYADMF11C1* | 243470 | R | Forward Primer | CTCCTGTGCCACCTTCTTATG |
| 14 | *MYADMF11C1* | 243470 | R | Reverse Primer | GCAGGCGGTGGCAAA |
| 14 | *MYADMF11C1* | 243470 | R | Vic Probe | CTCTCAGGCTCCATC |
| 14 | *MYADMF11C1* | 243470 | R | Fam Probe | CTCTCAGGCTCCGTC |
| 15 | *MYADMF11C1* | 243590 | R | Forward Primer | CAGGAGGCGCAGGTGATG |
| 15 | *MYADMF11C1* | 243590 | R | Reverse Primer | CTTCTCCCTGGTGGCTGGT |
| 15 | *MYADMF11C1* | 243590 | R | Vic Probe | ATGATGAGGGTCATGAC |
| 15 | *MYADMF11C1* | 243590 | R | Fam Probe | ATGATGAGGGTCACGAC |
| 16 | *MYADMF11D1* | 266279 | Y | Forward Primer | GCCAGTTGTTGGTCCCAGAT |
| 16 | *MYADMF11D1* | 266279 | Y | Reverse Primer | TGGCCGCTCTACCAGTTCA |
| 16 | *MYADMF11D1* | 266279 | Y | Vic Probe | TCACTGGACCACTGG |
| 16 | *MYADMF11D1* | 266279 | Y | Fam Probe | CGTCACTGGACCGCT |
| 17 | *MYADMF11D1* | 266287 | M | Forward Primer | GGCCGCTCTACCAGTTCAA |
| 17 | *MYADMF11D1* | 266287 | M | Reverse Primer | GTTGTTGGTCCCAGATGCA |
| 17 | *MYADMF11D1* | 266287 | M | Vic Probe | TTGGGCAGGCCGC |
| 17 | *MYADMF11D1* | 266287 | M | Fam Probe | TTGGGCAGGCAGC |
| 18 | *MYADMF11D1* | 266381 | Y | Forward Primer | AGGACGGAGAGCAGGGTCA |
| 18 | *MYADMF11D1* | 266381 | Y | Reverse Primer | GCTGCCCATCCCCTTCT |
| 18 | *MYADMF11D1* | 266381 | Y | Vic Probe | CACAACCTGGAAAA |
| 18 | *MYADMF11D1* | 266381 | Y | Fam Probe | CACAACCTGGAGAA |
| 19 | *MYADMF11D1* | 266384 | R | Forward Primer | CCAGAGGAGCAGAGCAGTGG |
| 19 | *MYADMF11D1* | 266384 | R | Reverse Primer | ATCCTGGTGAACCTGGGCA |
| 19 | *MYADMF11D1* | 266384 | R | Vic Probe | CATCCCCTTCTCCATT |
| 19 | *MYADMF11D1* | 266384 | R | Fam Probe | CATCCCCTTCTCCGTT |
| 20 | *MYADMF11D1* | 266687 | R | Forward Primer | CCGTCATCTACATTCAGTTCTTGT |
| 20 | *MYADMF11D1* | 266687 | R | Reverse Primer | TCACGTCCATGGCATAAGC |
| 20 | *MYADMF11D1* | 266687 | R | Vic Probe | CTGGGACCGCCCCA |
| 20 | *MYADMF11D1* | 266687 | R | Fam Probe | ACTGGGACCGCACC |
| 21 | *MYADMF11D1* | 266699 | R | Forward Primer | AGGTCACGTCCATGGCATAAG |
| 21 | *MYADMF11D1* | 266699 | R | Reverse Primer | CCCGTCATCTACATTCAGTTCTTG |
| 21 | *MYADMF11D1* | 266699 | R | Vic Probe | TCCCAGTTGTGGCTG |
| 21 | *MYADMF11D1* | 266699 | R | Fam Probe | TCCCAGTCGTGGCT |
| 22 | *MYADMF11D1* | 266710 | Y | Forward Primer | AGGTCACGTCCATGGCATAAG |
| 22 | *MYADMF11D1* | 266710 | Y | Reverse Primer | ATCTACCCCGTCATCTACATTCAGT |
| 22 | *MYADMF11D1* | 266710 | Y | Vic Probe | TGGCTGTTGAACAAG |
| 22 | *MYADMF11D1* | 266710 | Y | Fam Probe | TGGCTGTTGGACAAG |
| 23 | Peak SNP | 273823 | R | Forward Primer | CCAGGTAGGAAAGTTGCAGTATGC |
| 23 | Peak SNP | 273823 | R | Reverse Primer | GCCACCAGCACACACTTGC |
| 23 | Peak SNP | 273823 | R | Vic Probe | CTCCCTCTCTTTCTG |
| 23 | Peak SNP | 273823 | R | Fam Probe | CTCCCTCTCTTTTTG |
| 24 | Peak SNP | 286172 | Y | Forward Primer | GAGGCGCAGGTGATGAGAA |
| 24 | Peak SNP | 286172 | Y | Reverse Primer | TGGTGGCTGGTGTGAGCA |
| 24 | Peak SNP | 286172 | Y | Vic Probe | ATGATGAGGGTCACGAC |
| 24 | Peak SNP | 286172 | Y | Fam Probe | ATGATGAGGGTCATGAC |
| 25 | Peak SNP | 313303 | Y | Forward Primer | AGGACGGAGAGCAGGGT |
| 25 | Peak SNP | 313303 | Y | Reverse Primer | CTGGGCAAATGCGACAACAG |
| 25 | Peak SNP | 313303 | Y | Vic Probe | CCCATCCCCTTCTCCGTT |
| 25 | Peak SNP | 313303 | Y | Fam Probe | CCATCCCCTTCTCCATT |
| 26 | Outer Peak | 327664 | K | Forward Primer | ACGTTAGCAGGCATTTCTGTCT |
| 26 | Outer Peak | 327664 | K | Reverse Primer | AGCTGGGCTCTGTTTGCT |
| 26 | Outer Peak | 327664 | K | Vic Probe | CTGGATCTTAATTTCTG |
| 26 | Outer Peak | 327664 | K | Fam Probe | CTGGATCTTACTTTCTG |

**Table S3: Genomic Regions Associated with Intracellular RBC Potassium (RBC K)**

| ***SNP*** | ***CHR*** | ***Position*** | ***Best Fitting Model*** | ***Nominal P-value*** | ***Effect Size*** | ***Genes within 100 kb either side*** |
| --- | --- | --- | --- | --- | --- | --- |
| oar3_OAR18_19267427 | 18 | 19267427 | Genotypic | 1.11E-63 | 2141.5 | MYADMF11B1 (ENSOARG00000011091), MYADMF1G1 (ENSOARG00000011097), MYADMF1F1 (ENSOARG00000017376), MYADMF11A1 (ENSOARG00000017388), MYADMF1A2 (ENSOARG00000017401), MYADMF1A3 (ENSOARG00000017413), MYADMF1H1 (ENSOARG00000011119) |
| oar3_OAR18_19342055 | 18 | 19342055 | Genotypic | 1.11E-63 | 2141.5 | MYADMF1A3 (ENSOARG00000017413), MYADMF1H1 (ENSOARG00000011119), MYADMF1A5 (ENSOARG00000011124), MYADMF11D1 (ENSOARG00000017428) |
| oar3_OAR18_19354404 | 18 | 19354404 | Genotypic | 1.11E-63 | 2141.5 | MYADMF1A3 (ENSOARG00000017413), MYADMF1H1 (ENSOARG00000011119), MYADMF1A5 (ENSOARG00000011124), MYADMF11D1 (ENSOARG00000017428) |
| oar3_OAR18_19381535 | 18 | 19381535 | Genotypic | 1.11E-63 | 2141.5 | MYADMF1A3 (ENSOARG00000017413), MYADMF1H1 (ENSOARG00000011119), MYADMF1A5 (ENSOARG00000011124), MYADMF11D1 (ENSOARG00000017428) |
| oar3_OAR18_19324759 | 18 | 19324759 | Genotypic | 1.58E-63 | 2142.4 | MYADMF11A1 (ENSOARG00000017388), MYADMF1A2 (ENSOARG00000017401), MYADMF1A3 (ENSOARG00000017413), MYADMF1H1 (ENSOARG00000011119), MYADMF1A5 (ENSOARG00000011124), MYADMF11D1 (ENSOARG00000017428) |
| oar3_OAR18_19336673 | 18 | 19336673 | Genotypic | 4.74E-63 | 2141.1 | MYADMF1A3 (ENSOARG00000017413), MYADMF1H1 (ENSOARG00000011119), MYADMF1A5 (ENSOARG00000011124), MYADMF11D1 (ENSOARG00000017428) |
| oar3_OAR18_19226336 | 18 | 19226336 | Genotypic | 1.79E-45 | 1990.6 | MYADMF11B1 (ENSOARG00000011091), MYADMF1G1 (ENSOARG00000011097), MYADMF1F1 (ENSOARG00000017376), MYADMF11A1 (ENSOARG00000017388), MYADMF1A2 (ENSOARG00000017401), MYADMF1A3 (ENSOARG00000017413), MYADMF1H1 (ENSOARG00000011119) |
| oar3_OAR18_19395896 | 18 | 19395896 | Genotypic | 1.78E-29 | 2167.8 | MYADMF1H1 (ENSOARG00000011119), MYADMF1A5 (ENSOARG00000011124), MYADMF11D1 (ENSOARG00000017428) |
| oar3_OAR18_19399557 | 18 | 19399557 | Genotypic | 3.05E-26 | 1955.3 | MYADMF1H1 (ENSOARG00000011119), MYADMF1A5 (ENSOARG00000011124), MYADMF11D1 (ENSOARG00000017428) |
| oar3_OAR18_19190606 | 18 | 19190606 | Genotypic | 1.05E-21 | 1788.1 | MYADMF2C1 (ENSOARG00000011064), MYADMF11B1 (ENSOARG00000011091), MYADMF1G1 (ENSOARG00000011097), MYADMF1F1 (ENSOARG00000017376), MYADMF11A1 (ENSOARG00000017388), MYADMF1A2 (ENSOARG00000017401), MYADMF1A3 (ENSOARG00000017413) |
| oar3_OAR18_19242324 | 18 | 19242324 | Genotypic | 4.85E-19 | 1872.5 | MYADMF11B1 (ENSOARG00000011091), MYADMF1G1 (ENSOARG00000011097), MYADMF1F1 (ENSOARG00000017376), MYADMF11A1 (ENSOARG00000017388), MYADMF1A2 (ENSOARG00000017401), MYADMF1A3 (ENSOARG00000017413), MYADMF1H1 (ENSOARG00000011119) |
| oar3_OAR18_19250221 | 18 | 19250221 | Genotypic | 2.26E-12 | 1657.3 | MYADMF11B1 (ENSOARG00000011091), MYADMF1G1 (ENSOARG00000011097), MYADMF1F1 (ENSOARG00000017376), MYADMF11A1 (ENSOARG00000017388), MYADMF1A2 (ENSOARG00000017401), MYADMF1A3 (ENSOARG00000017413), MYADMF1H1 (ENSOARG00000011119) |
| oar3_OAR18_19254774 | 18 | 19254774 | Genotypic | 2.26E-12 | 1657.3 | MYADMF11B1 (ENSOARG00000011091), MYADMF1G1 (ENSOARG00000011097), MYADMF1F1 (ENSOARG00000017376), MYADMF11A1 (ENSOARG00000017388), MYADMF1A2 (ENSOARG00000017401), MYADMF1A3 (ENSOARG00000017413), MYADMF1H1 (ENSOARG00000011119) |
| oar3_OAR3_131511529 | 3 | 131511529 | Recessive | 2.80E-10 | 1604.8 | METAP2, USP44, ENSOARG00000016026 |
| oar3_OAR16_60932124 | 16 | 60932124 | Recessive, Genotypic | 1.58E-09 | 1485.3 | None |
| oar3_OAR9_33886033 | 9 | 33886033 | Genotypic | 2.26E-09 | 1980.1 | PCMTD1, ST18 |
| oar3_OAR18_19384885 | 18 | 19384885 | Allelic | 3.22E-09 | 2130.5 | MYADMF1H1 (ENSOARG00000011119), MYADMF1A5 (ENSOARG00000011124), MYADMF11D1 (ENSOARG00000017428) |
| oar3_OAR18_19507995 | 18 | 19507995 | Recessive | 4.70E-09 | 969.7 | ACAN, HAPLN3, MFGE8 |
| oar3_OAR1_224100316 | 1 | 224100316 | Recessive | 4.72E-09 | 1110.8 | SPTSSB |
| oar3_OAR3_56656271 | 3 | 56656271 | Genotypic | 5.27E-09 | 1693.2 | KCMF1 |
| oar3_OAR18_18035615 | 18 | 18035615 | Genotypic | 5.65E-09 | 1928.7 | ENSOARG00000010826 |
| OAR18_39083150.1 | 18 | 36936501 | Genotypic | 1.04E-08 | 1183.3 | None |
| oar3_OAR12_66279215 | 12 | 66279215 | Recessive | 2.48E-08 | 1642.2 | PLA2G4A |
| oar3_OAR16_4995853 | 16 | 4995853 | Genotypic | 3.79E-08 | 2102.3 | NKX2-5, STC2 |
| oar3_OAR1_137738754 | 1 | 137738754 | Allelic | 3.99E-08 | 2224.5 | C21orf91, BTG3, ENSOARG00000015844 |
| oar3_OAR1_193170747 | 1 | 193170747 | Allelic | 4.09E-08 | 2153.75 | FGF12 |
|  |  |  |  |  |  |  |

**Table S4: Genomic Regions Associated with Intracellular RBC Sodium (RBC Na)**

| ***SNP*** | ***CHR*** | ***Position*** | ***Best Fitting Model*** | ***Nominal P-value*** | ***Effect Size*** | ***Genes within 100 kb either side*** |
| --- | --- | --- | --- | --- | --- | --- |
| oar3_OAR18_19324759 | 18 | 19324759 | Genotypic | 7.27E-33 | 1604.3 | MYADMF11A1 (ENSOARG00000017388), MYADMF1A2 (ENSOARG00000017401), MYADMF1A3 (ENSOARG00000017413), MYADMF1H1 (ENSOARG00000011119), MYADMF1A5 (ENSOARG00000011124), MYADMF11D1 (ENSOARG00000017428) |
| oar3_OAR18_19342055 | 18 | 19342055 | Genotypic | 1.46E-32 | 1601.8 | MYADMF1A3 (ENSOARG00000017413), MYADMF1H1 (ENSOARG00000011119), MYADMF1A5 (ENSOARG00000011124), MYADMF11D1 (ENSOARG00000017428) |
| oar3_OAR18_19267427 | 18 | 19267427 | Genotypic | 1.46E-32 | 1601.8 | MYADMF11B1 (ENSOARG00000011091), MYADMF1G1 (ENSOARG00000011097), MYADMF1F1 (ENSOARG00000017376), MYADMF11A1 (ENSOARG00000017388), MYADMF1A2 (ENSOARG00000017401), MYADMF1A3 (ENSOARG00000017413), MYADMF1H1 (ENSOARG00000011119) |
| oar3_OAR18_19354404 | 18 | 19354404 | Genotypic | 1.46E-32 | 1601.8 | MYADMF1A3 (ENSOARG00000017413), MYADMF1H1 (ENSOARG00000011119), MYADMF1A5 (ENSOARG00000011124), MYADMF11D1 (ENSOARG00000017428) |
| oar3_OAR18_19381535 | 18 | 19381535 | Genotypic | 1.46E-32 | 1601.8 | MYADMF1A3 (ENSOARG00000017413), MYADMF1H1 (ENSOARG00000011119), MYADMF1A5 (ENSOARG00000011124), MYADMF11D1 (ENSOARG00000017428) |
| oar3_OAR18_19336673 | 18 | 19336673 | Genotypic | 2.74E-32 | 1452.2 | MYADMF1A3 (ENSOARG00000017413), MYADMF1H1 (ENSOARG00000011119), MYADMF1A5 (ENSOARG00000011124), MYADMF11D1 (ENSOARG00000017428) |
| oar3_OAR18_19226336 | 18 | 19226336 | Recessive | 6.53E-28 | 1486 | MYADMF11B1 (ENSOARG00000011091), MYADMF1G1 (ENSOARG00000011097), MYADMF1F1 (ENSOARG00000017376), MYADMF11A1 (ENSOARG00000017388), MYADMF1A2 (ENSOARG00000017401), MYADMF1A3 (ENSOARG00000017413), MYADMF1H1 (ENSOARG00000011119) |
| oar3_OAR18_19395896 | 18 | 19395896 | Genotypic | 6.67E-20 | 1588.5 | MYADMF1H1 (ENSOARG00000011119), MYADMF1A5 (ENSOARG00000011124), MYADMF11D1 (ENSOARG00000017428) |
| oar3_OAR18_19399557 | 18 | 19399557 | Genotypic | 1.08E-18 | 1452.2 | MYADMF1H1 (ENSOARG00000011119), MYADMF1A5 (ENSOARG00000011124), MYADMF11D1 (ENSOARG00000017428) |
| oar3_OAR18_19190606 | 18 | 19190606 | Genotypic | 6.34E-16 | 1333.8 | MYADMF2C1 (ENSOARG00000011064), MYADMF11B1 (ENSOARG00000011091), MYADMF1G1 (ENSOARG00000011097), MYADMF1F1 (ENSOARG00000017376), MYADMF11A1 (ENSOARG00000017388), MYADMF1A2 (ENSOARG00000017401), MYADMF1A3 (ENSOARG00000017413) |
| oar3_OAR18_19242324 | 18 | 19242324 | Genotypic | 6.72E-15 | 1412.6 | MYADMF11B1 (ENSOARG00000011091), MYADMF1G1 (ENSOARG00000011097), MYADMF1F1 (ENSOARG00000017376), MYADMF11A1 (ENSOARG00000017388), MYADMF1A2 (ENSOARG00000017401), MYADMF1A3 (ENSOARG00000017413), MYADMF1H1 (ENSOARG00000011119) |
| oar3_OAR18_19250221 | 18 | 19250221 | Genotypic | 1.07E-10 | 1284.9 | MYADMF11B1 (ENSOARG00000011091), MYADMF1G1 (ENSOARG00000011097), MYADMF1F1 (ENSOARG00000017376), MYADMF11A1 (ENSOARG00000017388), MYADMF1A2 (ENSOARG00000017401), MYADMF1A3 (ENSOARG00000017413), MYADMF1H1 (ENSOARG00000011119) |
| oar3_OAR18_19254774 | 18 | 19254774 | Genotypic | 1.07E-10 | 1284.9 | MYADMF11B1 (ENSOARG00000011091), MYADMF1G1 (ENSOARG00000011097), MYADMF1F1 (ENSOARG00000017376), MYADMF11A1 (ENSOARG00000017388), MYADMF1A2 (ENSOARG00000017401), MYADMF1A3 (ENSOARG00000017413), MYADMF1H1 (ENSOARG00000011119) |
| oar3_OAR18_19507995 | 18 | 19507995 | Recessive | 3.40E-08 | 738.5 | ACAN, HAPLN3, MFGE8 |
| oar3_OAR3_56656271 | 3 | 56656271 | Genotypic | 3.52E-08 | 1221.4 | KCMF1 |
| oar3_OAR16_60932124 | 16 | 60932124 | Recessive | 4.82E-08 | 1153.1 | None |

**Table S5: Genomic Regions Associated with Intracellular RBC Sodium/Potassium Ratio (RBC Na/K)**

| ***SNP*** | ***CHR*** | ***Position*** | ***Best Fitting Model*** | ***Nominal P-value*** | ***Effect Size*** | ***Genes within 100 kb either side*** |
| --- | --- | --- | --- | --- | --- | --- |
| oar3_OAR18_19324759 | 18 | 19324759 | Genotypic | 2.77E-21 | 6.4 | MYADMF11A1 (ENSOARG00000017388), MYADMF1A2 (ENSOARG00000017401), MYADMF1A3 (ENSOARG00000017413), MYADMF1H1 (ENSOARG00000011119), MYADMF1A5 (ENSOARG00000011124), MYADMF11D1 (ENSOARG00000017428) |
| oar3_OAR18_19267427 | 18 | 19267427 | Genotypic | 6.39E-21 | 6.4 | MYADMF11B1 (ENSOARG00000011091), MYADMF1G1 (ENSOARG00000011097), MYADMF1F1 (ENSOARG00000017376), MYADMF11A1 (ENSOARG00000017388), MYADMF1A2 (ENSOARG00000017401), MYADMF1A3 (ENSOARG00000017413), MYADMF1H1 (ENSOARG00000011119) |
| oar3_OAR18_19342055 | 18 | 19342055 | Genotypic | 6.39E-21 | 6.4 | MYADMF1A3 (ENSOARG00000017413), MYADMF1H1 (ENSOARG00000011119), MYADMF1A5 (ENSOARG00000011124), MYADMF11D1 (ENSOARG00000017428) |
| oar3_OAR18_19354404 | 18 | 19354404 | Genotypic | 6.39E-21 | 6.4 | MYADMF1A3 (ENSOARG00000017413), MYADMF1H1 (ENSOARG00000011119), MYADMF1A5 (ENSOARG00000011124), MYADMF11D1 (ENSOARG00000017428) |
| oar3_OAR18_19381535 | 18 | 19381535 | Genotypic | 6.39E-21 | 6.4 | MYADMF1A3 (ENSOARG00000017413), MYADMF1H1 (ENSOARG00000011119), MYADMF1A5 (ENSOARG00000011124), MYADMF11D1 (ENSOARG00000017428) |
| oar3_OAR18_19336673 | 18 | 19336673 | Genotypic | 1.01E-20 | 6.4 | MYADMF1A3 (ENSOARG00000017413), MYADMF1H1 (ENSOARG00000011119), MYADMF1A5 (ENSOARG00000011124), MYADMF11D1 (ENSOARG00000017428) |
| oar3_OAR18_19226336 | 18 | 19226336 | Genotypic | 6.46E-20 | 6.1 | MYADMF11B1 (ENSOARG00000011091), MYADMF1G1 (ENSOARG00000011097), MYADMF1F1 (ENSOARG00000017376), MYADMF11A1 (ENSOARG00000017388), MYADMF1A2 (ENSOARG00000017401), MYADMF1A3 (ENSOARG00000017413), MYADMF1H1 (ENSOARG00000011119) |
| oar3_OAR18_19399557 | 18 | 19399557 | Genotypic | 3.39E-16 | 6.1 | MYADMF1H1 (ENSOARG00000011119), MYADMF1A5 (ENSOARG00000011124), MYADMF11D1 (ENSOARG00000017428) |
| oar3_OAR18_19395896 | 18 | 19395896 | Allelic | 1.07E-15 | 6.2 | MYADMF1H1 (ENSOARG00000011119), MYADMF1A5 (ENSOARG00000011124), MYADMF11D1 (ENSOARG00000017428) |
| oar3_OAR18_19242324 | 18 | 19242324 | Genotypic | 2.81E-13 | 6 | MYADMF11B1 (ENSOARG00000011091), MYADMF1G1 (ENSOARG00000011097), MYADMF1F1 (ENSOARG00000017376), MYADMF11A1 (ENSOARG00000017388), MYADMF1A2 (ENSOARG00000017401), MYADMF1A3 (ENSOARG00000017413), MYADMF1H1 (ENSOARG00000011119) |
| oar3_OAR18_19190606 | 18 | 19190606 | Genotypic | 7.70E-12 | 5.1 | MYADMF2C1 (ENSOARG00000011064), MYADMF11B1 (ENSOARG00000011091), MYADMF1G1 (ENSOARG00000011097), MYADMF1F1 (ENSOARG00000017376), MYADMF11A1 (ENSOARG00000017388), MYADMF1A2 (ENSOARG00000017401), MYADMF1A3 (ENSOARG00000017413) |
| oar3_OAR18_19250221 | 18 | 19250221 | Allelic | 1.70E-12 | 5.7 | MYADMF11B1 (ENSOARG00000011091), MYADMF1G1 (ENSOARG00000011097), MYADMF1F1 (ENSOARG00000017376), MYADMF11A1 (ENSOARG00000017388), MYADMF1A2 (ENSOARG00000017401), MYADMF1A3 (ENSOARG00000017413), MYADMF1H1 (ENSOARG00000011119) |
| oar3_OAR18_19254774 | 18 | 19254774 | Allelic | 1.70E-12 | 5.7 | MYADMF11B1 (ENSOARG00000011091), MYADMF1G1 (ENSOARG00000011097), MYADMF1F1 (ENSOARG00000017376), MYADMF11A1 (ENSOARG00000017388), MYADMF1A2 (ENSOARG00000017401), MYADMF1A3 (ENSOARG00000017413), MYADMF1H1 (ENSOARG00000011119) |
| oar3_OAR3_56656271 | 3 | 56656271 | Genotypic | 1.72E-08 | 3.9 | KCMF1 |
| oar3_OAR14_47612540 | 14 | 47612540 | Genotypic | 1.91E-08 | 6 | RYR1, MAP4K1, EIF3K, ACTN4, CAPN12, ENSOARG00000005829, ENSOARG00000005822, ENSOARG00000026925 |
| oar3_OAR1_252246975 | 1 | 52246975 | Dominant | 1.99E-08 | 5.2 | None |
| oar3_OAR16_46184453 | 16 | 46184453 | Allelic | 2.80E-08 | 4 | None |
| oar3_OAR18_19507995 | 18 | 19507995 | Recessive | 3.00E-08 | 3.7 | ACAN, HAPLN3, MFGE8 |
| oar3_OAR1_133357515 | 1 | 133357515 | Allelic, Dominant | 3.14E-08 | 4.8 | None |

**Table S10: Genetic Variants within MYADMF ORFs**

| ***BAC position (bp)*** | ***Position in ORF*** | ***In Manual and Automated List*** | ***NT in ref.*** | ***NT in alt.*** | ***AA Ref*** | ***AA Alt*** |
| --- | --- | --- | --- | --- | --- | --- |
| **175021** | **257** | **Y** | **C** | **T** | **Glycine** | **Glutamic acid** |
| **194258** | **828** | **N** | **C** | ***** | **stop two AA away** | **Arginine** |
| **194262** | **824** | **N** | **G** | **A** | **Serine** | **Phenylalanine** |
| **194267** | **819** | **N** | **T** | **C** | **Leucine** | **Leucine** |
| **194268** | **818** | **N** | **G** | **A** | **Serine** | **Leucine** |
| **194288** | **798** | **N** | **G** | **A** | **Tyrosine** | **Tyrosine** |
| **194305** | **781** | **Y** | **T** | **C** | **Isoleucine** | **Valine** |
| **194351** | **735** | **Y** | **A** | **T** | **Serine** | **Arginine** |
| **194355** | **731** | **Y** | **A** | **T** | **Phenylalanine** | **Tyrosine** |
| **194369** | **717** | **Y** | **G** | **A** | **Serine** | **Serine** |
| **194373** | **713** | **Y** | **A** | **G** | **valine** | **alanine** |
| **194460** | **626** | **N** | **A** | **G** | **valine** | **alanine** |
| **194470** | **616** | **N** | **C** | **T** | **Valine** | **methionine** |
| **194471** | **615** | **N** | **A** | **G** | **Threonine** | **Threonine** |
| **194490** | **596** | **Y** | **C** | **T** | **Arginine** | **histidine** |
| **194492** | **594** | **Y** | **G** | **A** | **Serine** | **Serine** |
| **194510** | **576** | **Y** | **A** | **G** | **Aspartic acid** | **aspartic acid** |
| **194524** | **562** | **Y** | **A** | **G** | **Tryptophan** | **arginine** |
| **194554** | **532** | **Y** | **A** | **T** | **Phenylalanine** | **Isoleucine** |
| **194557** | **529** | **Y** | **G** | **A** | **Leucine** | **Phenylalanine** |
| **194574** | **512** | **N** | **C** | **G** | **Glycine** | **Alanine** |
| **194587** | **499** | **N** | **C** | **G** | **Valine** | **Leucine** |
| **194594** | **492** | **N** | **C** | **T** | **Proline** | **Proline** |
| **194605** | **481** | **N** | **T** | **G** | **Methionine** | **Leucine** |
| **194610** | **476** | **N** | **G** | **A** | **Proline** | **Leucine** |
| **194613** | **473** | **N** | **A** | **G** | **Phenylalanine** | **Serine** |
| **194622** | **464** | **N** | **A** | **C** | **Isoleucine** | **Serine** |
| **194654** | **432** | **N** | **G** | **C** | **Isoleucine** | **methionine** |
| **194662** | **424** | **N** | **A** | **G** | **Leucine** | **Leucine** |
| **194664** | **422** | **N** | **C** | **T** | **Arginine** | **Lysine** |
| **194669** | **417** | **N** | **C** | **G** | **Leucine** | **Leucine** |
| **194726** | **360** | **Y** | **A** | **T** | **Aspartic acid** | **Glutamic acid** |
| **194744** | **342** | **Y** | **A** | **T** | **Serine** | **Serine** |
| **194854** | **232** | **Y** | **T** | **C** | **Isoleucine** | **Valine** |
| **194866** | **220** | **Y** | **G** | **A** | **Leucine** | **Phenylalanine** |
| **212948** | **16** | **Y** | **A** | **G** | **Threonine** | **Alanine** |
| **212976** | **44** | **Y** | **G** | **T** | **Arginine** | **methionine** |
| **212980** | **48** | **Y** | **A** | **T** | **Threonine** | **Threonine** |
| **213128** | **196** | **Y** | **A** | **G** | **Isoleucine** | **Valine** |
| **213186** | **254** | **Y** | **A** | **T** | **Tyrosine** | **Phenylalanine** |
| **213187** | **255** | **N** | **T** | **C** | **Phenylalanine** | **Phenylalanine** |
| **213273** | **341** | **Y** | **A** | **G** | **glutamine** | **Arginine** |
| **213286** | **354** | **Y** | **C** | **T** | **Isoleucine** | **Isoleucine** |
| **213293** | **361** | **Y** | **A** | **G** | **Threonine** | **Alanine** |
| **213298** | **366** | **Y** | **A** | **G** | **Alanine** | **Alanine** |
| **213310** | **378** | **Y** | **T** | **C** | **Isoleucine** | **Isoleucine** |
| **213430** | **GCM** | **N** | **A** | **C** | **Alanine** | **Alanine** |
| **213494** | **562** | **Y** | **A** | **T** | **Serine** | **Cysteine** |
| **213551** | **619** | **Y** | **C** | **A** | **Proline** | **Threonine** |
| **213552** | **620** | **N** | **T** | **C** | **Methionine** | **Threonine** |
| **213563** | **631** | **Y** | **A** | **G** | **Lysine** | **Glutamic acid** |
| **213669** | **737** | **Y** | **A** | **G** | **asparagine** | **Serine** |
| **213670** | **738** | **N** | **T** | **C** | **Serine** | **Serine** |
| **213696** | **764** | **Y** | **A** | **G** | **glutamine** | **Arginine** |
| **213722** | **790** | **Y** | **G** | **A** | **glutamic acid** | **lysine** |
| **213764** | **832** | **N** | **G** | **A** | **valine** | **methionine** |
| **213770** | **838** | **Y** | **G** | **A** | **valine** | **Isoleucine** |
| **213779** | **847** | **Y** | **A** | **G** | **Threonine** | **Alanine** |
| **213793** | **861** | **Y** | **G** | **A** | **Leucine** | **Leucine** |
| **213794** | **862** | **N** | **A** | **G** | **Isoleucine** | **Valine** |
| **213824** | **892** | **Y** | **T** | **C** | **Phenylalanine** | **Leucine** |
| **213825** | **893** | **N** | **G** | **T** | **Arginine** | **Leucine** |
| **213839** | **907** | **Y** | **G** | **A** | **glutamic acid** | **Lysine** |
| **224546** | **254** | **Y** | **G** | **A** | **Threonine** | **Isoleucine** |
| **224550** | **250** | **Y** | **C** | **T** | **Valine** | **Isoleucine** |
| **224557** | **243** | **Y** | **G** | **A** | **Aspartic acid** | **aspartic acid** |
| **224585** | **215** | **Y** | **G** | **A** | **Proline** | **Leucine** |
| **224633** | **167** | **Y** | **T** | **C** | **Tyrosine** | **Cysteine** |
| **224799** | **1** | **Y** | **C** | **T** | **Valine** | **methionine** |
| **242861** | **859** | **Y** | **G** | **A** | **glutamine** | **stop** |
| **242895** | **825** | **N** | **C** | **A** | **Valine** | **Valine** |
| **243040** | **680** | **Y** | **T** | **C** | **Tyrosine** | **Cysteine** |
| **243110** | **NA** | **N** | **GG** | **GGGGGACG** | **NA** | **NA** |
| **243168** | **552** | **Y** | **T** | **G** | **Alanine** | **Alanine** |
| **243213** | **507** | **Y** | **A** | **C** | **Leucine** | **Leucine** |
| **243266** | **454** | **Y** | **C** | **T** | **valine** | **Isoleucine** |
| **243267** | **453** | **N** | **A** | **G** | **cysteine** | **cysteine** |
| **243276** | **444** | **Y** | **A** | **G** | **Phenylalanine** | **Phenylalanine** |
| **243296** | **424** | **Y** | **G** | **A** | **Leucine** | **Phenylalanine** |
| **243467** | **253** | **Y** | **C** | **T** | **Valine** | **Isoleucine** |
| **243470** | **250** | **Y** | **T** | **C** | **Isoleucine** | **Valine** |
| **243590** | **130** | **Y** | **C** | **T** | **valine** | **methionine** |
| **266238** | **756** | **Y** | **G** | **A** | **Serine** | **Serine** |
| **266279** | **715** | **Y** | **G** | **A** | **Arginine** | **tryptophan** |
| **266287** | **707** | **Y** | **T** | **G** | **Glutamine** | **Proline** |
| **266343** | **651** | **Y** | **G** | **A** | **Tyrosine** | **Tyrosine** |
| **266369** | **625** | **Y** | **G** | **A** | **Leucine** | **Leucine** |
| **266381** | **613** | **Y** | **G** | **A** | **Leucine** | **Phenylalanine** |
| **266384** | **610** | **Y** | **C** | **T** | **Valine** | **Isoleucine** |
| **266466** | **528** | **Y** | **G** | **A** | **Tyrosine** | **Tyrosine** |
| **266472** | **522** | **Y** | **G** | **A** | **Alanine** | **Alanine** |
| **266504** | **490** | **Y** | **G** | **A** | **Leucine** | **Leucine** |
| **266652** | **342** | **Y** | **G** | **A** | **Phenylalanine** | **Phenylalanine** |
| **266667** | **TTY** | **Y** | **G** | **A** | **Phenylalanine** | **Phenylalanine** |
| **266687** | **307** | **Y** | **G** | **T** | **Proline** | **Threonine** |
| **266699** | **295** | **Y** | **C** | **T** | **Aspartic acid** | **asparagine** |
| **266710** | **284** | **N** | **G** | **A** | **Serine** | **Phenylalanine** |
| **266751** | **243** | **Y** | **C** | **T** | **Serine** | **Serine** |

**Table S11: Human MYADM and KCC3A predicted interacting residues**

MYADM

I25

N66

W67

F70

A92

P95

C185

F188

P194

N195

Y197

Q198

P201

E204

V207

A208

A211

I212

F214

I215

I219

L222

G226

E227

C228

N230

V231

L232

P233

I234

KCC3A

R143

P144

V146

S147

L150

N151

M153

A154

I295

R298

K342

F343

S345

L346

A349

C350

V353

F367

A368

F372

E395

K556

N558

W566

P567

S568

W570

V571

I574

**Table S12: Ovine MYADM and KCC3A predicted interacting residues**

MYADM

F51

A55

S56

W60

N66

M69

F70

I84

G88

L90

R93

P95

S192

R281

H283

KCC3A

S147

L150

R298

Y339

K342

K364

K394

V396

W566

P567

S568

P569

W570

I574

**Table S10: Predicted interacting residues for ovine Texel v4 reference or alternate allele MYADMF1G1 and KCC3A**

MYADMF1G1_Texel v4_Reference

F39

A43

D44

I47

Y51

N54

W55

M57

S58

C61

F62

I69

T72

L75

R76

L78

F82

F84

L88

S181

N182

T183

L187

W217

L272

R316

KCC3A (interacting with MYADMF1G1 Texel v4_Reference)

E90

D91

S147

L150

S151

A154

V296

R298

H303

I335

Y339

K342

F343

L346

H371

K394

P567

S568

W570

V571

I574

F578

MYADMF1G1_Alternate

C22

P24

C27

L29

H33

N34

M36

A41

Y79

N82

W83

S86

S108

P111

F112

W114

K180

T181

G201

F204

S208

N209

T210

Y213

L214

H215

P217

E220

V223

A224

S227

F230

I231

L235

L238

L239

N240

E243

W244

R247

KCC3A (interacting with MYADMF1G1_Alternate)

L133

E137

E138

M140

T142

P144

K145

V146

S147

L149

L150

S151

L153

A154

R298

K342

S345

L346

A349

C350

V353

K364

F367

A368

P370

H371

F372

K556

N558

W566

P567

S568

P569

W570

V571

I574

**Table S13: Predicted interacting residues for ovine reference or alternate MYADMF1B3 and KCC3A**

MYADMF1B3_Texel v4_Reference

T10

S14

D18

A19

S22

F26

L29

P30

F33

S34

V37

S40

V42

F47

Q48

S61

I62

T65

I69

S71

I110

T111

Y112

V113

Q114

V116

P117

G119

P120

D123

R124

A127

G175

I177

L181

KCC3A (interacting with MYADMF1B3 Texel v4_Reference)

L308

S311

A312

L315

R319

K454

D480

I481

T482

P606

L632

L635

I636

L641

I645

F649

Y701

Y702

V705

I709

M712

Y716

Y719

I730

Second KCC3A in dimer interacting with MYADMF1B3_Texel_v4_Reference

L218

Q219

T444

L447

W448

Y451

H680

W681

A682

F685

M686

S689

MYADMF1B3_Alternate

N108

W109

M111

P112

F113

C115

V116

F136

F138

E208

G229

F232

S236

T238

Y241

L242

E248

V251

A252

S255

I256

F258

L259

V263

L266

L267

A270

E271

W272

Y274

M275

F283

KCC3A (interacting with MYADMF1B3_Alternate)

P144

V146

S147

L149

L150

L153

A154

F188

S345

L346

A349

C350

V353

I363

K364

F367

A368

H371

F372

K556

N558

W566

P567

S568

W570

V571

I574
